# Elucidating mechano-pathology of osteoarthritis: transcriptome-wide differences in mechanically stressed aged human cartilage explants

**DOI:** 10.1186/s13075-021-02595-8

**Published:** 2021-08-16

**Authors:** Evelyn Houtman, Margo Tuerlings, Janne Riechelman, Eka H. E. D. Suchiman, Robert J. P. van der Wal, Rob G. H. H. Nelissen, Hailiang Mei, Yolande F. M. Ramos, Rodrigo Coutinho de Almeida, Ingrid Meulenbelt

**Affiliations:** 1 Section of Molecular Epidemiology, Department of Biomedical Data Sciences, Leiden University Medical Center, Postzone S05-P, Einthovenweg 20, 2333 ZC Leiden, The Netherlands; 2 Department of Orthopaedics, Leiden University Medical Center, Leiden, The Netherlands; 3 Sequencing Analysis Support Core, Leiden University Medical Centre, Leiden, The Netherlands

**Keywords:** Osteoarthritis, Cartilage, Chondrocytes, Mechanical stress, Mechano-pathology, RNA sequencing, Cellular senescence, IGF-1 signaling, MMP13

## Abstract

**Background:**

Failing of intrinsic chondrocyte repair after mechanical stress is known as one of the most important initiators of osteoarthritis. Nonetheless, insight into these early mechano-pathophysiological processes in age-related human articular cartilage is still lacking. Such insights are needed to advance clinical development. To highlight important molecular processes of osteoarthritis mechano-pathology, the transcriptome-wide changes following injurious mechanical stress on human aged osteochondral explants were characterized.

**Methods:**

Following mechanical stress at a strain of 65% (65%MS) on human osteochondral explants (*n*_65%MS_ = 14 versus *n*_control_ = 14), RNA sequencing was performed. Differential expression analysis between control and 65%MS was performed to determine mechanical stress-specific changes. Enrichment for pathways and protein-protein interactions was analyzed with Enrichr and STRING.

**Results:**

We identified 156 genes significantly differentially expressed between control and 65%MS human osteochondral explants. Of note, *IGFBP5* (FC = 6.01; FDR = 7.81 × 10^−3^) and *MMP13* (FC = 5.19; FDR = 4.84 × 10^−2^) were the highest upregulated genes, while *IGFBP6* (FC = 0.19; FDR = 3.07 × 10^−4^) was the most downregulated gene. Protein-protein interactions were significantly higher than expected by chance (*P* = 1.44 × 10^−15^ with connections between 116 out of 156 genes). Pathway analysis showed, among others, enrichment for cellular senescence, insulin-like growth factor (IGF) I and II binding, and focal adhesion.

**Conclusions:**

Our results faithfully represent transcriptomic wide consequences of mechanical stress in human aged articular cartilage with *MMP13*, IGF binding proteins, and cellular senescence as the most notable results. Acquired knowledge on the as such identified initial, osteoarthritis-related, detrimental responses of chondrocytes may eventually contribute to the development of effective disease-modifying osteoarthritis treatments.

**Supplementary Information:**

The online version contains supplementary material available at 10.1186/s13075-021-02595-8.

## Introduction

Osteoarthritis (OA) is an age-related joint disease, affecting diarthrodial joints [1, 2]. Despite the fact that OA is the most prevalent and disabling disease among elderly, resulting in high social and economic burden, no effective treatment exists except for lifestyle changes, pain medication, and eventually a joint replacement surgery at end-stage disease [3, 4].

To characterize deregulated signaling pathways in OA cartilage, comprehensive differential expression analyses have been performed comparing preserved versus end-stage lesioned OA cartilage [5]. These studies revealed that OA pathology is marked by recuperation of growth plate signaling, wound healing, and skeletal system development, while also highlighting inherent differences in OA pathophysiology between patient subtypes based on gene expression changes [5–7]. Nonetheless, the preserved versus lesioned study design by definition captures end-stage pathophysiological OA disease processes and gives no information on early initial processes triggering cartilage to become diseased. In contrast, disease-modifying OA drugs should preferably target early OA disease triggers when irreversible damage of cartilage has not yet taken place. Therefore, more knowledge on the initial response of chondrocytes to OA-relevant stresses, such as mechanical trauma, should be investigated in an appropriate model.

In this regard, failing of intrinsic chondrocyte repair after mechanical stress is known to impact the integrity of articular cartilage via cell apoptosis [8], increased catabolic gene expression [9], and reduced matrix production [10] and is, as such, an important trigger to OA onset. Nonetheless, little knowledge exists on the inherent dysregulation of signaling pathways initiating repair responses in human aged articular cartilage upon mechanical stress. To gain some insight, several in vivo animal studies have investigated the effect of joint overuse or trauma on gene expression in cartilage [11–16]. Some examples of non-invasive in vivo mechanical loading studies are Bomer et al. [11], reporting on involvement of metabolic processes and skeletal system development pathways upon physiological forced running in 6-month-old mice; Chang et al. [14], reporting on involvement of cell proliferation and chondroitin sulfate proteoglycan metabolic process upon injurious tibial compression in 16-week-old mice; and Sebastian et al. [13], reporting on single-cell RNA-seq upon tibial compression in 10-week-old mice. Thus far, one study has investigated genome-wide expression consequences of an impact injury in porcine explants and identified involvement of genes associated with matrix molecules, protein biosynthesis, skeletal development, and cell proliferation [17]. Nevertheless, most studies were performed using relatively young animal tissues and likely do not cover the biological response to a trauma in adult (human) tissue [18]. More recently, global gene expression profiling in 14-month-old mice subjected to non-invasive injurious tibial compression identified genes involved in inflammation and matrix regeneration to be involved in the response of aged tissue [14].

A more appropriate model to identify which molecular processes are initiated in response to mechanical stress in humans would comprise aged human ex vivo osteochondral explants. Injurious compression reaching strains above 50% induced catabolic processes in cartilage and eventually led to cell death [19]. In aged human osteochondral explants, injurious cyclic mechanical stress at a strain of 65% (65%MS), mimicking trauma, was previously shown to induce OA-like damage [20]. In the current study, we therefore exploited our previously established ex vivo osteochondral explant model by performing RNA sequencing on explants subjected to injurious mechanical stress in comparison to controls. The hypothesis-free, transcriptome-wide approach presented here contributes to further understanding the debilitating response of aged chondrocytes to mechanical injury and how this affects their propensity to enter an OA disease state.

## Material and methods

### Sample description

To generate osteochondral explants, biopsies (diameter of 8 mm) were punched from the macroscopically preserved load-bearing area of femoral condyles of human knee joints obtained within the Research in Articular Osteoarthritis Cartilage (RAAK) biobank containing patients that undergo a joint replacement surgery as a consequence of OA [21]. For this study, a total of 60 osteochondral explants were investigated originating from nineteen independent donors in which multiple explants were taken from each donor. This difference between the amount of samples taken per donor was dependent on several factors. Among them were the size of knee condyle, size of the preserved area, surgical damage area, and other simultaneous experiments this donor was used for. RNA sequencing was performed on samples from nine donors, while the remaining ten donors were used for replication purposes. All donor characteristics are given in Table S1 and were equal between mechanical stressed and control explant donors.

### Application of mechanical stress

**Fig. 1:**
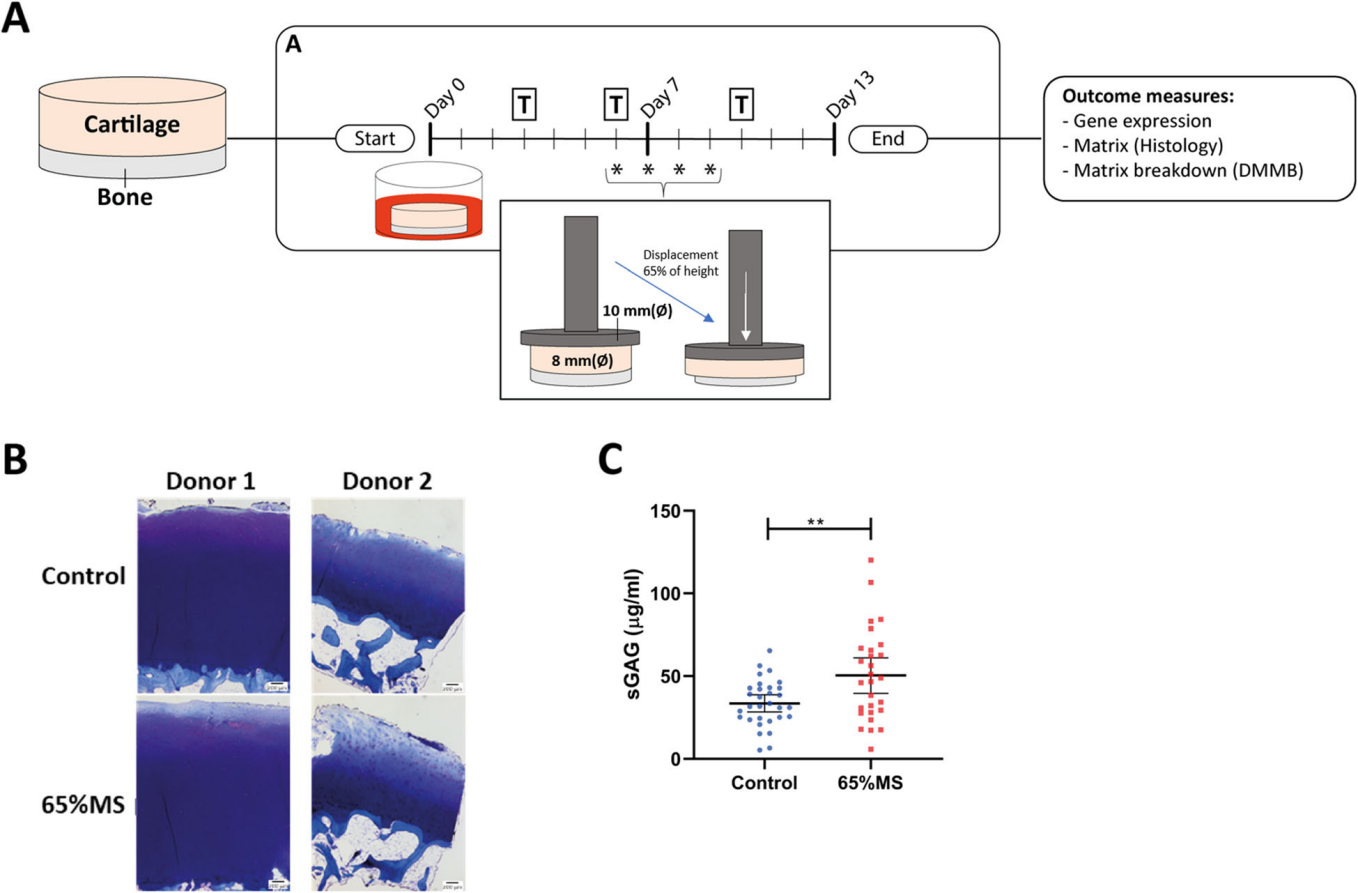
Study setup of human osteochondral explants receiving 65% MS. **A** Osteochondral explants were punched from preserved areas of knee joints and the medium is refreshed on indicated days (T). **B** Damage in our mechanical stress model was confirmed by degradation of sGAG in cartilage by toluidine blue staining (histology of two independent donors) and measuring **C** sGAG release in conditioned media on day 13 (*n*_control_ = 31 versus *n*_65%MS_ = 28). The average ± 95% CI are presented with each dot representing a sample. To adjust for donor variation, *P*-values were estimated by performing logistic generalized estimation equations, with sGAG concentration as dependent variable and treatment as covariate: *sGAG concentration ∼ Treatment + (1|Donor)*. **P ≤ 0.01. Legend: 65%MS 65% mechanical stress, DMMB dimethylmethylene blue, sGAG sulfated glycosaminoglycans

Explants of nineteen donors were equilibrated in serum-free chondrogenic differentiation medium (DMEM, supplemented with ascorbic acid (50 μg/ml; Sigma-Aldrich; Zwijndrecht, The Netherlands), L-proline (40 μg/ml; Sigma-Aldrich), sodium pyruvate (100 μg/ml; Sigma-Aldrich), dexamethasone (0.1 μM; Sigma-Aldrich), ITS+, and antibiotics (100 U/ml penicillin; 100 μg/ml streptomycin)) in a 5% (v/v) CO2 incubator at 37 °C. As depicted in Fig. 1A, after a 6-day period, dynamic unconfined compression was applied to explants (diameter of 8 mm) using the Mach-1 mechanical testing system on 4 subsequent days (Biomomentum Inc., Laval, QC, Canada). In short, osteochondral explants were placed under an indenter (diameter of 10 mm) attached to a 250-N MACH-1 load cell (Fig. 1A) and unconfined cyclic compression was applied at a strain of 65% of cartilage height at a frequency of 1 Hz (1 compression cycle per second), mimicking walking speed, during 10 min, long enough to be injurious and short enough for chondrocytes to survive, at strains suggested to be detrimental [22]. Dynamic (cyclic) compression means that a force was applied that varied over time to simulate a more cyclic compression such as walking. To investigate lasting effects of mechanical stress, 4 days after mechanical stress, the cartilage and bone were separated, snap-frozen in liquid nitrogen, and stored at −80 °C.

### Determining cartilage integrity

#### Histology

A sagittal section of the osteochondral explant was fixed in 4% formaldehyde for 1 week and decalcified using EDTA (12.5%, pH = 7.4) during 2 weeks, dehydrated with an automated tissue processing apparatus, and embedded in paraffin. Tissue sections were cut at a thickness of 5 μm, deparaffinized, rehydrated, subsequently stained for 1 min in a toluidine blue solution with a pH of 2.5 (Sigma-Aldrich), and mounted with Pertex (Sigma-Aldrich) to investigate cartilage integrity as quantified by applying Mankin Score [23].

#### Sulfated glycosaminoglycan (sGAG) measurement

Sulfated glycosaminoglycan (sGAG) concentrations in conditioned media collected from osteochondral explants were measured with the photometric 1.9 dimethylene blue (DMMB; Sigma-Aldrich) dye method [24]. Shark chondroitin sulfate (Sigma-Aldrich) was used as the reference standard. The concentration of sGAG was determined in conditioned media collected on day 13, by measuring absorbance at 525 nm and 595 nm in a microplate reader (Synergy HT; BioTek, Winooski, USA).

## RNA sequencing

RNA from cartilage was extracted by pulverizing the tissue and subsequently homogenizing the powder in TRIzol reagent (Invitrogen, San Diego, CA) using a Mixer mill 200 (Retsch, Germany). RNA was extracted using chloroform, followed by precipitation using ethanol, and purified with the RNeasy Mini Kit (Qiagen, Chatsworth, CA). Genomic DNA was removed by DNase digestion. Paired-end 2 × 150 base pair RNA sequencing (Illumina TruSeq mRNA Library Prep Kit, Illumina HiSeq X ten) was performed. Strand-specific RNA-sequencing libraries were generated which yielded on average 14 million reads per sample. Data from the Illumina platform was analyzed with an in-house pipeline as previously described [5]. The adapters were clipped using Cutadapt v1.1. RNA-seq reads were then aligned using GSNAP against GRCh38 [25]. Read abundances per sample were estimated using HTSeq count v0.11.1 [26] with Ensembl gene annotation version 94. Only uniquely mapping reads were used for estimating expression. The quality of the raw reads and initial processing for RNA sequencing was checked using MulitQC v1.7 [27]. Samples containing > 50% genes with zero values and average read count < 10 were removed from further analysis. To identify outliers, principal component analysis (PCA) was applied. For further analysis, samples not in the main cluster were removed, resulting in *n* = 28 samples from 9 unique donors. In total, 58,735 unique genes were detected by RNA sequencing of which 6509 were protein-coding genes that were included in further analyses.

### Differential expression analysis, protein-protein interactions, and pathway enrichment

Differential expression analysis was performed in 65%MS cartilage compared to control cartilage obtained from osteochondral explants using DESeq2 R package version 1.24 [28] on 6509 protein-coding genes. A general linear model assuming a negative binominal distribution was applied and followed by a Wald test between control and 65%MS samples in which donor number was added as a random effect to correct for inter-individual differences. In all analyses, control samples were set as reference. To correct for multiple testing, the Benjamini-Hochberg method was used, as indicated by the false discovery rate (FDR) in which a significant cutoff value of 0.05 was used. Furthermore, the comprehensive gene set enrichment analysis web tool Enrichr [29] was used to identify enrichment for gene ontologies (Cellular Component, Biological Process, Molecular Function) and pathways (KEGG and Reactome). For protein-protein interactions, analysis was performed using the online tool STRING version 11.0 [30].

### Real-time quantitative PCR (RT-qPCR) validation

250 ng of RNA was processed into cDNA using the First Strand cDNA Synthesis Kit (Roche Applied Science, Almere, The Netherlands). RT-qPCR was performed on 10 paired 65%MS samples with matched controls included in the RNA sequencing (Technical validation) and 10 novel paired 65%MS samples with matched controls (Biological validation) to determine the expression of six downregulated (*IGFBP6*, *CNTFR*, *WISP2*, *FRZB*, *COL9A3*, and *GADD45A*) and four upregulated genes (*IGFBP5*, *PTGES*, *TNC*, and *IGFBP4*)*.* Primer sequences are listed in Table S2. The relative gene expression was normalized for two endogenous reference genes, *SDHA* and *YWHAZ*, to determine −ΔCT values. To determine effect sizes, fold changes (FC) were calculated according to the 2^−ΔΔCT^ method, in which expression of 65%MS was extracted from controls (−ΔΔCT). These two endogenous reference genes were chosen based on literature stating the stability of these genes in response to mechanical stress, which was confirmed by our RNA sequencing [31, 32].

### Statistical analysis

Analysis on RNA-sequencing data was performed in R as described above. Statistical analysis for RT-qPCR and sGAG concentrations were performed using IBM SPSS statistics 25. The *P*-values were determined by applying a linear generalized estimating equation (GEE) to effectively adjust for dependencies among donors of the explants by adding a random effect for the sample donor as we did not have perfect pairs for each analysis [33]. The following GEE was fitted in which gene expression was the dependent variable and treatment the covariate: *Gene expression ~ Treatment + (1|donor)*. To determine differences in sGAG concentration on day 13, another linear GEE model was fitted with sGAG concentration as dependent variable and treatment as covariate: *sGAG concentration ~ Treatment + (1|donor)*.

## Results

Prior to RNA sequencing, cartilage tissue integrity of human osteochondral explants was characterized by performing histology and measuring sGAG concentrations in conditioned media. Mechanical strains at 65% cause detrimental changes to cartilage integrity as previously shown [20] (Fig. 1B), and these effects were further explored in a larger samples size (*n*_control_ = 31; *n*_65%MS_ = 28), where an increased sGAG release was measured in 65%MS cartilage when compared to controls (Fig. 1C).

### Differential expression of genes responsive to injurious mechanical stress

**Fig. 2:**
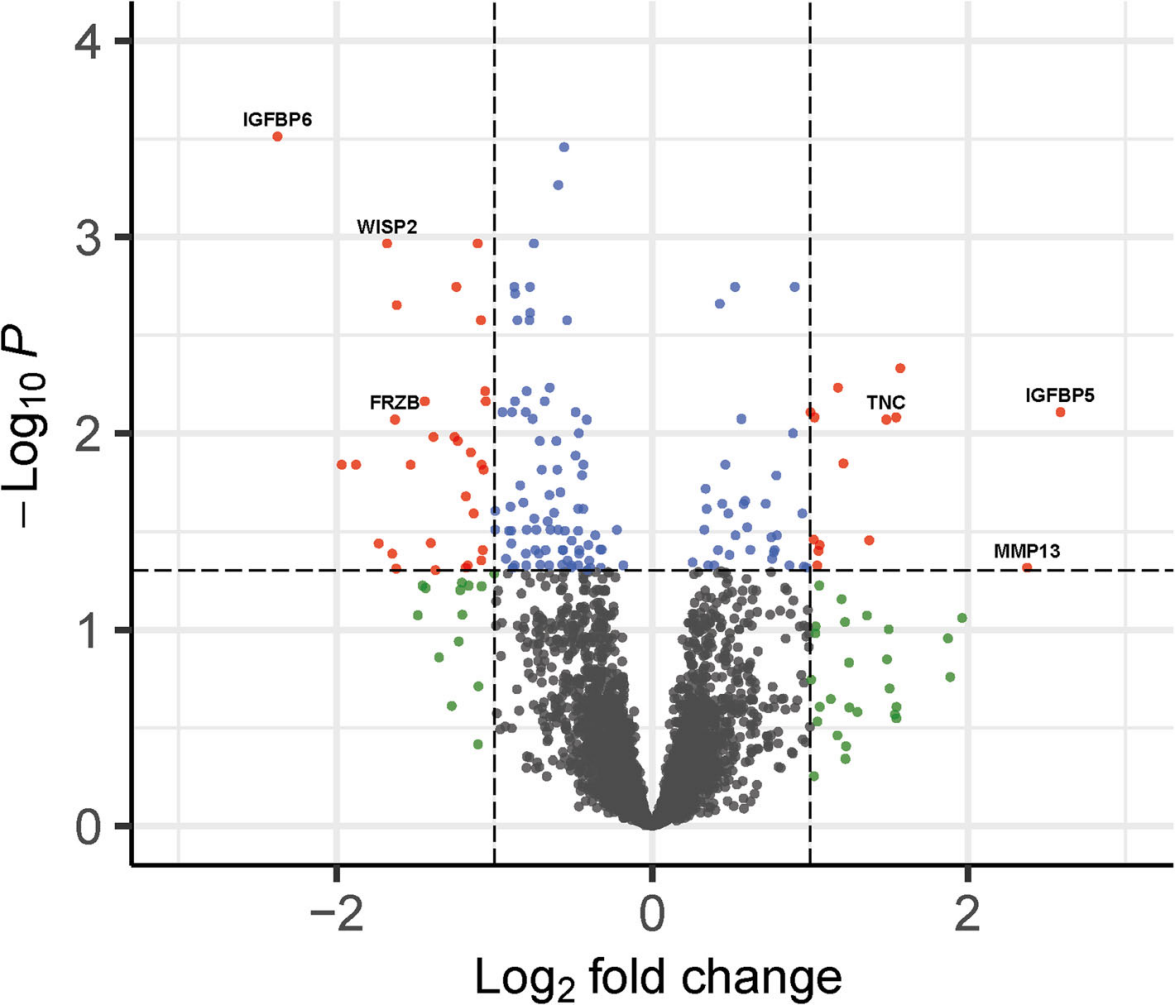
Volcano plot of differentially expressed genes. Dots represent genes expressed in mechanically stressed cartilage in comparison to control osteochondral explant cartilage. Red dots represent significantly differentially expressed (DE) genes that have an absolute fold change (FC) of ≥2, blue dots represent significantly DE genes, green dots represent genes that have an absolute FC of ≥2 but are not significantly DE, and gray dots represent genes not DE expressed between controls and 65% mechanically stressed cartilage. The FC presented here is the gene expression of 65% mechanically stressed relative to control cartilage

To characterize the response of cartilage to mechanical stress at a strain of 65% indentation in aged articular cartilage, we performed RNA sequencing on control (*n* = 14 samples) and 65% mechanically stressed (*n* = 14 samples) articular cartilage samples obtained from macroscopically preserved osteochondral explants of human patients that underwent a knee replacement surgery due to OA. Baseline characteristics of donors of the RNA-sequencing dataset are depicted in Table S1a. We found 156 genes to be significantly differentially expressed (DE) (FDR < 0.05) with absolute fold changes (FC) ranging between 1.1 and 6.0 (Fig. 2, Table S3). Among these 156 DE genes, 46 (29%) were upregulated and 110 (71%) were downregulated. The 20 genes with the highest absolute FC, and their respective direction of effect previously identified in OA cartilage [5], are shown in Table 1. Notable among the upregulated genes were *IGFBP5* (FC = 6.01; FDR = 7.81 × 10^−3^), *MMP13* (FC = 5.19; FDR = 4.84 × 10^−2^), *TNC* (FC = 2.80; FDR = 8.51 × 10^−3^), and *PTGES* (FC = 2.92; FDR = 8.29 × 10^−3^). Notable genes among the downregulated genes were *IGFBP6* (FC = 0.19; FDR = 3.07 × 10^−4^), *CNTFR* (FC = 0.27; FDR = 1.44 × 10^−2^), *WISP2* (FC = 0.31; FDR = 1.08 × 10^−3^), and *FRZB* (FC = 0.32; FDR = 8.51 × 10^−3^).

**Table 1:** Top 20 genes with the highest absolute FC in 65% mechanically stressed cartilage compared to controls

Ensemble ID	Gene name	FC	FDR^a^	Differential expression in OA cartilage [5]^b^
ENSG00000115461	*IGFBP5*	6.01	7.81 × 10^−3^	
ENSG00000137745	*MMP13*	5.19	4.84 × 10^−2^	
ENSG00000204103	*MAFB*	2.97	4.66 × 10^−3^	↓
ENSG00000148344	*PTGES*	2.92	8.29 × 10^−3^	↑
ENSG00000041982	*TNC*	2.80	8.51 × 10^−3^	↑
ENSG00000141753	*IGFBP4*	2.59	3.50 × 10^−2^	↑
ENSG00000160111	*CPAMD8*	0.39	4.98 × 10^−2^	↓
ENSG00000166165	*CKB*	0.38	1.04 × 10^−2^	↑
ENSG00000106258	*CYP3A5*	0.38	3.62 × 10^−2^	
ENSG00000107736	*CDH23*	0.37	6.88 × 10^−3^	
ENSG00000187720	*THSD4*	0.35	1.44 × 10^−2^	
ENSG00000144908	*ALDH1L1*	0.33	2.22 × 10^−3^	↓
ENSG00000092758	*COL9A3*	0.32	4.89 × 10^−2^	
ENSG00000162998	*FRZB*	0.32	8.51 × 10^−3^	↓
ENSG00000170891	*CYTL1*	0.32	4.11 × 10^−2^	
ENSG00000064205	*WISP2*	0.31	1.08 × 10^−3^	↓
ENSG00000082196	*C1QTNF3*	0.30	3.64 × 10^−2^	↑
ENSG00000122756	*CNTFR*	0.27	1.44 × 10^−2^	↓
ENSG00000165966	*PDZRN4*	0.26	1.44 × 10^−2^	↓
ENSG00000167779	*IGFBP6*	0.19	3.07 × 10^−4^	

^a^To correct for multiple testing, the Benjamini-Hochberg method was applied to *P*-values and reported as the false discovery rate (FDR). ^b^Gene expression changes measured in RNA-sequencing data between preserved and lesioned OA articular cartilage, with preserved as reference [5]. Legend: *FC* fold change, *FDR* false discovery rate

### Validation of differentially expressed genes with mechanical stress

**Fig. 3:**
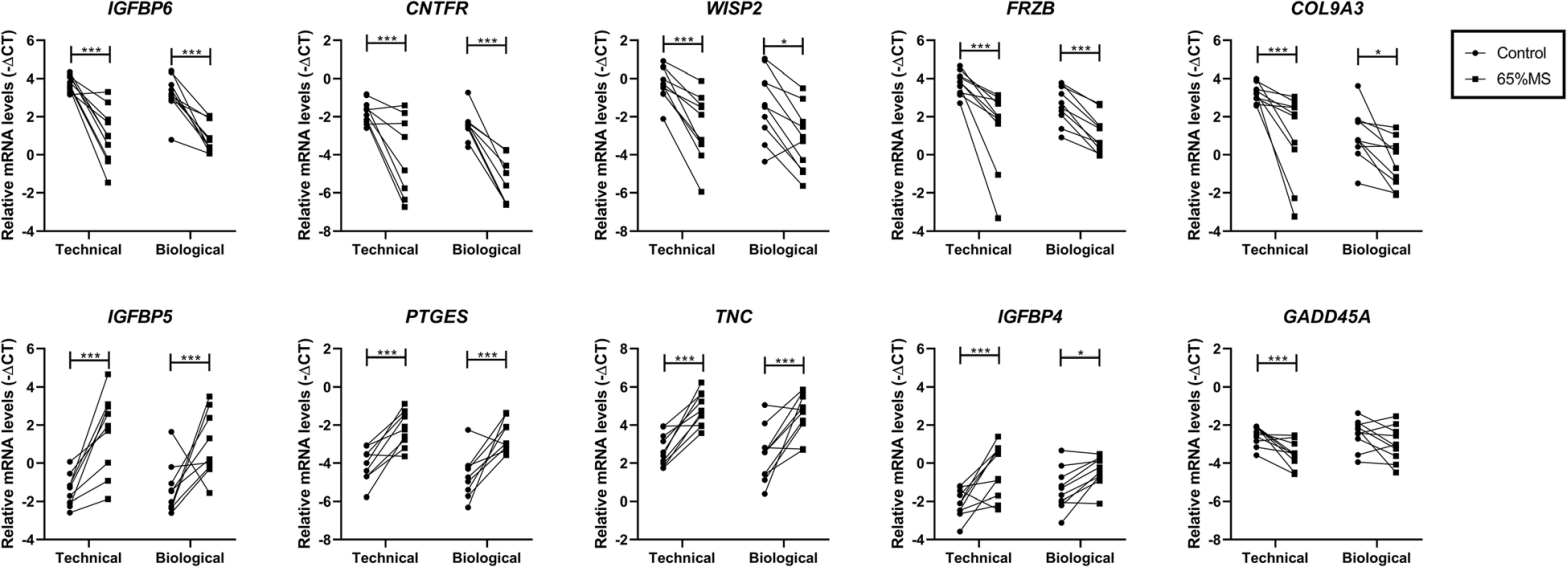
Technical and biological validation of the highest up- and downregulated genes was performed using RT-qPCR. Expression of six downregulated (*IGFBP6*, *CNTFR*, *WISP2*, *FRZB*, and *GADD45A*) and four upregulated (*IGFBP5*, *PTGES*, *TNC*, and *IGFBP4*) genes was measured in *n* = 10 paired technical and *n* = 10 paired biological osteochondral explants. Figures show connected paired samples and −ΔCT of each independent sample is depicted by black dots (control) or squares (65%MS) in the graphs. Statistical differences between gene expression in control and 65% mechanically stressed were determined with a linear generalized estimation equation (GEE) with mRNA level as the dependent variable. *P ≤ 0.05; ***P ≤ 0.001. Legend: 65%MS 65% mechanical stress, RT-qPCR reverse transcriptase-quantitative PCR

For validation and replication of the differentially expressed genes identified, a set of samples for technical (*n* = ten pairs) and biological (*n* = ten pairs) replication was selected for RT-qPCR. Baseline characteristics of donors in the replication dataset are depicted in Table S1b. Replication was performed for ten genes (Fig. 3), of which six were upregulated (*IGFBP6*, *CNTFR*, *WISP2*, *FRZB*, *COL9A3*, and *GADD45A*) and four were downregulated (*IGFBP5*, *PTGES*, *TNC*, and *IGFBP4*). Technical replication showed a significant difference for all ten genes between controls and 65%MS cartilage, with similar direction and size of effects. Biological replication also showed the same direction of effects and similar effect sizes as identified in the RNA-sequencing data. For *GADD45A*, however, the difference was not significant (*P*-value = 0.12). Taken together, technical and biological replication confirmed the robustness of our RNA-sequencing results.

### In silico exploration of differentially expressed genes

To explore whether significant DE genes (*N* = 156 genes) were involved in particular pathways, they were further analyzed using Enrichr. Gene enrichment was observed, among others, for insulin-like growth factor I and II binding (GO:0031995; GO:0031994, Padj = 1.83 × 10^−2^; Padj = 2.89 × 10^−2^, involving *IGFBP4*, *IGFBP5*, and *IGFBP6*), cellular senescence (hsa04218, Padj = 1.15 × 10^−2^, involving 8 genes, e.g., *GADD45A*, *MYC*, *SERPINE1*, and *FOXO1*), and focal adhesion (GO:0005925; hsa04510, Padj = 2.54 × 10^−2^; Padj = 1.33 × 10^−2^, involving 11 and 6 genes, respectively, e.g., *TNC*, *CAV1*, and *TLN2*) (Table 2; Table S4a).

**Table 2:** Gene ontology and pathway enrichment analysis of differentially expressed genes in mechanically stressed cartilage

Term	Entry	Overlap	Adj ***P***-value^**a**^	Odds ratio	Genes
Cellular senescence	hsa04218	8/160	1.15 × 10^−2^	6.41	GADD45A, MYC, SERPINE1, AKT3, EIF4EBP1, SLC25A5, ETS1, FOXO1
Focal adhesion	hsa04510	8/199	1.33 × 10^−2^	5.15	SHC4, CAV1, ITGA10, AKT3, LAMA3, TNC, COL9A3, TLN2
Insulin-like growth factor II binding	GO:0031995	3/7	1.83 × 10^−2^	54.95	IGFBP5, IGFBP4, IGFBP6
Focal adhesion	GO:0005925	11/356	2.54 × 10^−2^	3.96	ENAH, EHD3, GSN, CAV1, TNC, CD9, TLN2, RPL10A, DCAF6, RHOB, ENG
Insulin-like growth factor I binding	GO:0031994	3/13	2.89 × 10^−2^	29.59	IGFBP5, IGFBP4, IGFBP6

^a^Enrichr uses a modified Fisher’s exact test to compute enrichment and this is reported as the adjusted *P*-value [29]. Legend: *Adj P-value*, adjusted *P*-value

**Fig. 4:**
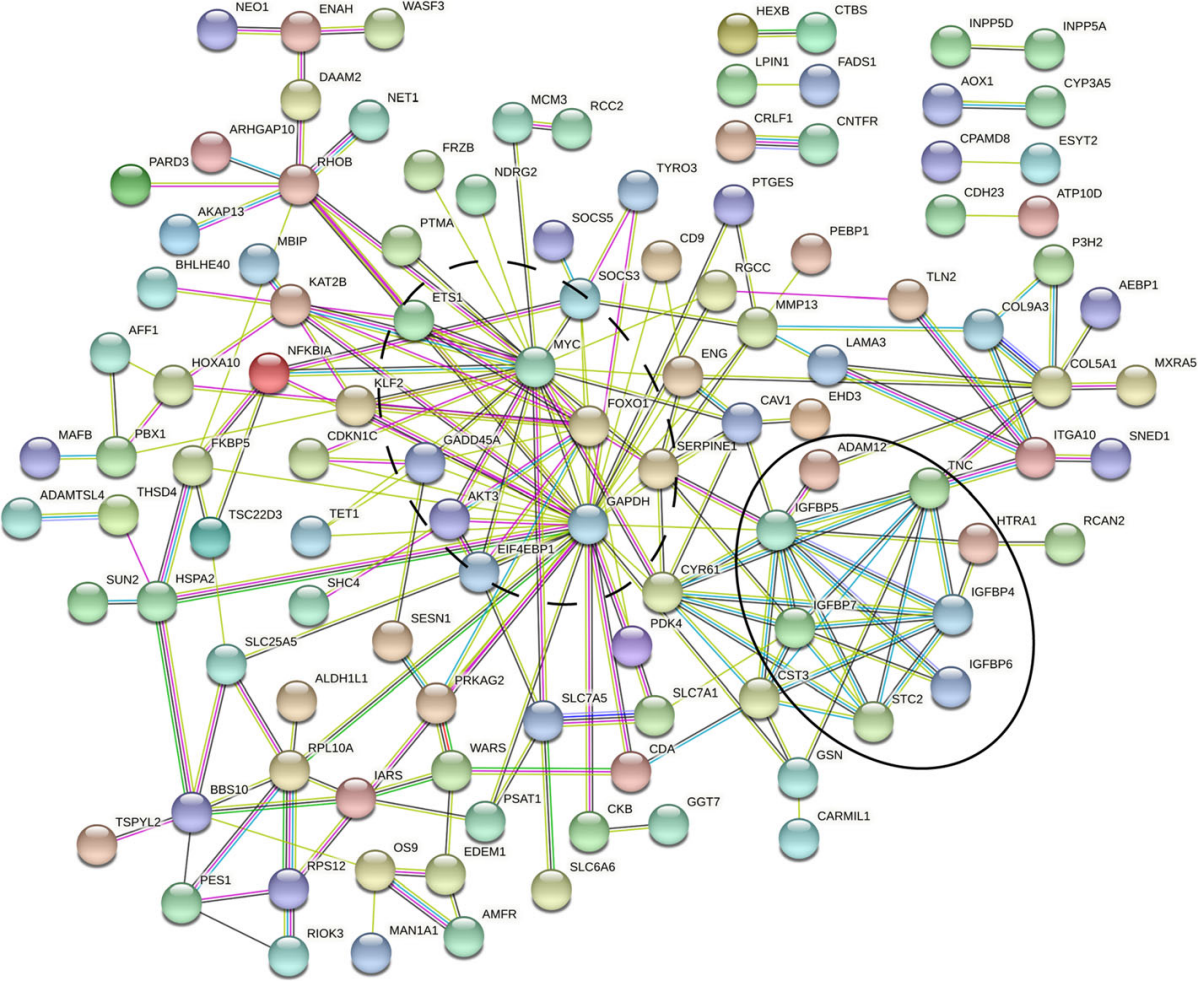
Protein-protein interaction network in STRING of proteins encoded by differentially expressed genes. Only connected (*N* = 116 genes) genes that were identified to be differentially expressed between mechanically stressed and control cartilage of osteochondral explants are shown. Two clusters with high interactions were identified upon studying connections within STRING. One cluster corresponds with genes found in the cellular senescence pathway (dotted circle), while the other cluster consists of proteins that are involved in IGF-1 signaling (black circle)

To visualize interacting proteins, the online tool STRING was used. Among the 156 genes, 116 of the encoded proteins showed significant protein-protein interactions (PPI) (*P* = 1.44 × 10^−15^; Fig. 4). Among these proteins, we found several that have many connections with other proteins in the DE gene network, such as *GAPDH* with 35 connections, *IGFBP5* with 12 connections, and in the cellular senescence involved genes *MYC* and *FOXO1* with respectively 26 and 13 connections to other DE genes. Moreover, two clusters of genes are observed that correspond with two of the pathways identified. One cluster corresponds with genes found mainly in the cellular senescence pathway (Fig. 4, dotted circle), while the other cluster consists of proteins that are involved in IGF-1 signaling (Fig. 4, black circle).

### Comparison between mechanical stress genes and OA-responsive genes

To investigate to what extend the genes DE with mechanical stress (DE_MS_) coincide with OA pathophysiology, we next compared the DE_MS_ genes (Table S3) to previously identified genes DE between preserved and lesioned OA cartilage (DE_OA_) [5]. Of the 156 DE_MS_ genes, 64 were previously identified with OA pathophysiology and their majority (48 genes, 75%) had the same direction of effect (Table 1 and Table S5a, Figure S1). Notable genes coinciding with OA pathophysiology and showing the same direction of effect are the highly downregulated *FRZB*, *WISP2*, and *CNTFR* and the upregulated *PTGES* and *CRLF1*.

Next, we selected for exclusive mechanical stress-responsive genes, i.e., DE_MS_ genes, not overlapping with previously identified DE_OA_ genes [5]. This resulted in 92 genes that were differentially expressed exclusively in response to mechanical stress (DE_ExclusiveMS_; Table S6; Figure S1). Notable DE_ExclusiveMS_ genes are the downregulated *IGFBP6*, *ITGA10*, and *COL9A3* and the upregulated *IGFBP5*, *MMP13*, and *GAPDH*. Subsequent pathway analyses showed gene enrichment among genes involved in focal adhesion (GO:0005925, Padj = 0.02, 9 genes, e.g., *CD9*, *RPL10A*, and *ENAH*) and kinase inhibitor activity (GO:0019210, Padj = 0.01, 5 genes, e.g., *CDKN1C*, *SOCS3*, and *SOCS5*) (Table S4b). Upon exploring protein-protein interactions between the 92 DE_ExclusiveMS_ genes using STRING, a highly significant enrichment for PPI was identified (*P* = 1.07 × 10^−4^; Figure S2), indicating that these genes act together or respond in concert to detrimental mechanical stress.

### OA risk genes responding to mechanical stress

Finally, to investigate which OA risk genes are represented among the mechanically stress-responsive genes in cartilage, we checked *N* = 90 genes previously recognized as strong OA risk genes [34] identified in recent genome-wide association studies (GWAS) [35, 36]. As shown in Table S7, two of our identified DE_MS_ genes were also shown to be an OA risk gene in previous studies. These genes were *TNC*, encoding for tenascin C, which was highly increased (FC = 2.80; FDR = 8.5 × 10^−3^) upon 65%MS and *SCUBE1*, encoding for signal peptide, CUB domain, and EGF-like domain-containing 1, which was decreased (FC = 0.53; FDR = 0.04) upon 65%MS.

## Discussion

To our knowledge, we are the first to report genome-wide differentially expressed mRNAs in articular cartilage following repeated exposure to 65% mechanical stress using a human ex vivo osteochondral explant model. Since injurious loading is considered a major trigger in the initiation of OA onset, the results presented in our manuscript contribute important insight into how injurious stress affects the propensity of aged human articular chondrocytes to lose their steady state towards a debilitating OA disease state. Notable genes identified were different members of the insulin-like growth factor I and II binding family (*IGFBP6*, *IGFBP5*, and *IGFBP4*) and the catabolic gene *MMP13*. Gene enrichment analyses showed that cellular senescence (*GADD45A*, *MYC*, *SERPINE1*, and *FOXO1*) and focal adhesion (*ITGA10*, *TLN2*, and *CAV1*) processes are significantly changing in articular cartilage with injurious loading. Together, identified genes and pathways facilitate clinical development by exploring ways to counteract these initial unbeneficial responses to injurious loading by supplementing or inhibiting of key genes. Moreover, we advocate that here identified specific responsive genes to injurious loading can function as sensitive markers facilitating the development of scientifically founded strategies with respect to preventive or curative exercise OA therapy among elderly.

Among the highest FDR significantly upregulated genes with 65% mechanical stress, we identified *MMP13*, encoding matrix metallopeptidase 13 (FC = 5.19; FDR = 4.84 × 10^−2^) [20]. MMP13 is involved in the detrimental breakdown of extracellular matrix in articular cartilage by cleaving, among others, collagen type II. Despite the well-known role of MMP13 in collagen type II breakdown, it should be noted that the *MMP13* gene is not found to be responsive with end-stage OA pathophysiology, i.e., not consistent and not among the genes highest differentially expressed between preserved and lesioned OA cartilage (Table 1) [5, 21, 37]. We therefore advocate that *MMP13* expression could specifically mark initial responses to cartilage damage and not that of a chronic degenerative OA disease state. Henceforth, abrogating the *MMP13* signaling shortly after an injurious cartilage event could prevent the detrimental downstream enzymatic breakdown of extracellular matrix proteins. Moreover, and as indicated above, *MMP13* may be a suitable candidate sensitively marking injurious loading of aged human articular cartilage independent of other physiological factors such as OA disease state.

Four out of seven members of the insulin growth factor binding proteins (*IGFBP4*, *IGFBP5*, *IGFBP6*, and *IGFBP7*; Table S8) were found to be FDR DE. IGFBP1-6 have an equal or greater affinity for binding IGF-1 when compared to IGF-1R; hence, most of IGF-1 in the body is bound to IGFBPs, antagonizing IGF-1 signaling [38–41]. On the other hand, IGFBP7 has a low affinity for IGF and therefore more likely affects cell metabolism via binding to activin A, influencing the growth-suppressing effects of TGF-β, and antagonizing bone morphogenetic protein (BMP) signaling [42, 43]. IGFBP4 and IGFBP5 can also function as a transporter and bring IGF-1 close to its receptor, where IGF-1 is released via cleavage by proteins such as pregnancy-associated plasma protein-A (PAPPA), HtrA Serine Peptidase 1 (HTRA1), and disintegrin and metalloproteinase domain-containing protein 12 (ADAM12) [44–46]. Additionally, notable in this respect is that three genes, *HTRA1*, *ADAM12*, and *STC2* [47], involved in IGF-1 cleavage were found among the FDR significant upregulated genes in our dataset (Table S3). IGFBPs can also affect cells via IGF-independent mechanisms. The most noteworthy IGF-independent mechanism is observed for the highly upregulated *IGFBP5*, being induction of cell proliferation and apoptosis [48, 49]. In summary, our data showed that, despite the fact that the mechanical stress applied affected cartilage integrity (Fig. 1), the upregulation of *IGFBP4* and *IGFBP5* in combination with the upregulation of its cleaving proteins might reflect an anabolic response of chondrocytes to initiate repair by increasing bio-availability of IGF-1. Two studies support our suggestion that IGF-1 signaling might be a beneficial anabolic response to mechanical stress. In an OA dog model, increasing intact IGFBP5 proteins resulted in increased IGF-1 levels and reduced destruction of cartilage [50]. While in a human explant model, addition of IGF-1 after mechanical stress increased *COL2A1* gene expression and slightly increased cell viability [51]. Our results in combination with those previously found suggest that addition of IGFBP4 and/or IGFBP5 would be an interesting therapy to further explore in combatting the catabolic response.

To identify upstream processes and to put our results in a broader perspective, we investigated connections between genes on the protein level in STRING (Fig. 4) and determined pathway enrichment (Table 2) of the differentially expressed genes. Based on this pathway analysis, we identified enrichment for proteins involved in cellular senescence. DE genes with mechanical stress in this pathway have already been linked to aging and OA, such as *GADD45A*, *SERPINE1*, *MYC*, and *FOXO1*. Notable are the two transcription factors, *MYC* and *FOXO1*, showing many connections to other proteins (Fig. 4) and previously shown to be dysregulated in OA chondrocytes [52, 53]. *FOXO1* is an essential mediator of cartilage growth and homeostasis and its expression is decreased in aged and OA cartilage [52]. In addition, *FOXO1* was shown to be an antagonist of *MYC* and prevents, among others, ROS production [54, 55]. Our results suggest that reduced expression of *FOXO1* could be one of the reasons for increased expression of *MYC*. As one of the known responses of chondrocytes to mechanical stress is ROS production, this would be a promising target to follow up on in future research. Next to genes in this pathway, lookup of our DE_MS_ genes in a proteomic atlas of senescence-associated secretory phenotype (SASP) identified 35 of our DE_MS_ genes to have previously been found in different senescent cells (Figure S3) [56]. Taken together, the upregulation of *MYC* in combination with upregulation of several important SASP protein markers suggests increased cellular damage is occurring upon mechanical stress likely driving cells to go into senescence. As cellular senescence is a factor that is thought to play a significant role in the OA pathophysiology, our model could provide more knowledge on how this pathway is involved in the onset of OA and how therapeutics could be used to minimize this response [57].

To investigate whether OA risk loci could confer risk via modifying the response to mechanical stress, we compared DE_MS_ genes to strong OA risk genes identified in the most recent GWAS [35, 36]. This resulted in the identification of two OA risk genes, *TNC* and *SCUBE1*, present in our dataset (Table S7). Based on allelic imbalanced expression and linkage disequilibrium, the *TNC* OA risk allele rs1330349-C, in high linkage disequilibrium with the transcript SNP rs2274836-T, appeared to act via decreasing expression of *TNC* [58]. For that matter, the observed high upregulation of *TNC* expression with mechanical stress (FC = 2.80; FDR = 8.51 × 10^−3^) as well as the previously observed upregulation with OA pathophysiology (FC = 1.41; FDR = 1.09 × 10^−2^) [5] is likely a beneficial response to rescue or maintain articular cartilage integrity. This is further confirmed by animal studies showing that the addition of exogenous TNC reduced cartilage degeneration and repaired cartilage [59, 60]. In contrast, for the intronic OA risk SNP located in the vicinity of *SCUBE1* (rs528981060), we were not able to determine a transcript proxy SNP; hence, potential AEI of *SCUBE1* could not be explored.

With regard to overlap with in vivo animal models, we compare our DE genes to those found in physiological [11], surgical destabilization of the medial meniscus (DMM) [18] and non-invasive tibial compression (TC) models [12, 14]. The most striking overlap in DE genes (46 genes) was found between our model and the non-invasive TC model using gene expression data of 14-month-old mice 1 week after injury. Among the overlapping genes, we confirmed the involvement of all *IGFBPs*, *HTRA1*, and *ADAM12* and of several OA-associated genes such as *FRZB*, *TNC*, and *SCUBE1* in both models [14]. As also shown by other studies [14, 18], age of animals used in these models can greatly influence results. This could also, next to a difference in species, partially explain why there is little overlap with other injurious mechanical stress studies.

A strength of our aged human ex vivo osteochondral model is that it allowed us to investigate the chondrocyte response to an OA-relevant trigger in its natural environment. In addition, our model comprises aged cartilage, which is likely more vulnerable to OA onset, and hence, results are relevant to the population at risk. Another strong point in our model is that we measure the changes in gene expression that are measured 4 days post-injury as such reflecting representative lasting changes in chondrocyte signaling rather than acute stress responses only. On the other hand, our data could facilitate treatment strategies, prior to irreversible damage of OA-affected cartilage. Some limitations of our study are the relatively low sample size of 14 explants per condition, hence limiting our power. As a result, we may have missed subtle gene expression changes in response to detrimental mechanical stress. Another point of our study to address is the heterogeneity of preserved cartilage collected from OA patients with Mankin scores ranging from 0 to 7. Although such heterogeneity may also have affected the power of our study, hence the total number of differentially expressed genes with injurious loading, we want to highlight that despite the differences in Mankin scores, we were able to consistently detect (at the genome-wide significant level) 156 differentially expressed genes reflecting strong and/or very consistent mechano-pathological processes triggered after mechanical stress. Moreover, due to the heterogeneity in eligible waste articular cartilage after joint replacement surgery (i.e., osteochondral explants), we were not able to generate a RNA-sequencing dataset of perfect control—mechanically stressed sample pairs. Henceforth, to adjust for dependencies among control and/or mechanically stressed samples, we added donor as a random effect during differential expression analyses. Adding to the validity of this approach was the fact that we successfully replicated expression changes for ten genes in ten novel independent perfectly paired samples. A final limitation of our study is that we have focused on exploring gene expression changes following mechanical stress and have not studied changes at the protein level. However, we advocate that chondrocyte signaling at the gene expression level is a more sensitive measure of underlying ongoing processes.

## Conclusions

To conclude, our results faithfully represent transcriptomic wide consequences of injurious loading in human aged articular cartilage with *MMP13*, IGF binding proteins, and cellular senescence as the most notable results. Since injurious loading is considered a major trigger of OA onset, these findings provide important insight into how injurious stress affects the propensity of aged human articular chondrocytes to lose their steady state towards a debilitating OA disease state. Exploring ways to counteract the initial unbeneficial responses to injurious loading may facilitate clinical development prior to the onset of irreversible damage. Moreover, we advocate that the here identified unique responsive genes to injurious loading, such as *MMP13*, can function as a sensitive marker to strategically develop preventive and/or curative exercise therapy for OA independent of other physiological factors. Preferably such an endeavor would exploit our established ex vivo osteochondral model while applying variable mechanical loading regimes.

## Supplementary Information

**Additional file 1.** Supplementary Tables and Figures. List of content: Supplementary Table S1. [a] Donor characteristics of samples for which RNA was sequenced. [b] Donor characteristics of independent samples used for replication of RNA-sequencing findings. Supplementary Table S2. Primer sequences used for replication and validation by RT-qPCR. Supplementary Table S3. Genes differentially expressed in 65%MS (DE_MS_) cartilage compared to control cartilage of human osteochondral explants. Supplementary Table S4. Gene enrichment found in Enrichr. Enrichment for [a] 156 DE_MS_ genes in and [b] 92 DE_ExclusiveMS_ for the gene ontology terms: biological process, molecular function and cellular component 2018, and pathways: KEGG 2019 human and reactome. Supplementary Table S5. DE_MS_ genes coinciding with previously reported DE genes in OA pathophysiology (DE_OA_). [a] DE_MS_ genes with same direction of effect as DE_OA_ genes. [b] DE_MS_ genes with opposite direction of effect as DE_OA_ genes. Supplementary Table S6. Exclusive mechanical response genes (DE_ExclusiveMS_). Supplementary Table S7. Previously reported OA risk loci present in our DE gene dataset. Supplementary Table S8. All insulin growth factor binding proteins (IGFBPs) and related DE genes identified in our analysis. Supplementary Figure S1. Venn diagram of coinciding genes between differentially expressed genes in mechanically stressed versus control cartilage from osteochondral explants (DE_MS_) and previously identified differentially expressed genes in preserved versus lesioned OA cartilage (DE_OA_). Supplementary Figure S2. Protein-protein interaction network in STRING of proteins encoded by differentially expressed genes (N = 92 genes) not coinciding with OA pathophysiology (DE_ExclusiveMS_). Supplementary Figure S3. Heat-map of proteins present in SASP.

## Data Availability

The list of all significantly affected genes is included in the Supplementary data (Table S3).
