# Supplementary material for: Elucidating mechano-pathology of osteoarthritis: transcriptome-wide differences in mechanically stressed aged human cartilage explants
Source: Arthritis Res Ther. 2021 Aug 16;23:215. doi: 10.1186/s13075-021-02595-8 (PMC8365911; doi:10.1186/s13075-021-02595-8)
Supplement: Supplementary file 1 — Additional file 1. Supplementary Tables and Figures. List of content: Supplementary Table S1. [a] Donor characteristics of samples for which RNA was sequenced. [b] Donor characteristics of independent samples used for replication of RNA-sequencing findings. Supplementary Table S2. Primer sequences used for replication and validation by RT-qPCR. Supplementary Table S3. Genes differentially expressed in 65%MS (DEMS) cartilage compared to control cartilage of human osteochondral explants. Supplementary Table S4. Gene enrichment found in Enrichr. Enrichment for [a] 156 DEMS genes in and [b] 92 DEExclusiveMS for the gene ontology terms: biological process, molecular function and cellular component 2018, and pathways: KEGG 2019 human and reactome. Supplementary Table S5. DEMS genes coinciding with previously reported DE genes in OA pathophysiology (DEOA). [a] DEMS genes with same direction of effect as DEOA genes. [b] DEMS genes with opposite direction of effect as DEOA genes. Supplementary Table S6. Exclusive mechanical response genes (DEExclusiveMS). Supplementary Table S7. Previously reported OA risk loci present in our DE gene dataset. Supplementary Table S8. All insulin growth factor binding proteins (IGFBPs) and related DE genes identified in our analysis. Supplementary Figure S1. Venn diagram of coinciding genes between differentially expressed genes in mechanically stressed versus control cartilage from osteochondral explants (DEMS) and previously identified differentially expressed genes in preserved versus lesioned OA cartilage (DEOA). Supplementary Figure S2. Protein-protein interaction network in STRING of proteins encoded by differentially expressed genes (N = 92 genes) not coinciding with OA pathophysiology (DEExclusiveMS). Supplementary Figure S3. Heat-map of proteins present in SASP. [file 13075_2021_2595_MOESM1_ESM.docx]

**Supplementary Tables & Figures**

**List of contents:**

**Supplementary Table S1. [a]** Donor characteristics of samples for which RNA was sequenced. **[b]** Donor characteristics of independent samples used for replication of RNA-sequencing findings**.**

**Supplementary Table S2. Primer sequences used for replication and validation by RT-qPCR.**

**Supplementary Table S3. Genes differentially expressed in 65%MS (DE_MS_) cartilage compared to control cartilage of human osteochondral explants.**

**Supplementary Table S4. Gene enrichment found in Enrichr.** Enrichment for **[a]** 156 DE_MS_ genes in and **[b]** 92 DE_ExclusiveMS_ for the gene ontology terms: biological process, molecular function and cellular component 2018, and pathways: KEGG 2019 human and reactome.

**Supplementary Table S5. DE_MS_ genes coinciding with previously reported DE genes in OA pathophysiology (DE_OA_). [a]** DE_MS_ genes with same direction of effect as DE_OA_ genes. **[b]** DE_MS_ genes with opposite direction of effect as DE_OA_ genes.

**Supplementary Table S6. Exclusive mechanical response genes (DE_ExclusiveMS_).**

**Supplementary Table S7. Previously reported OA risk loci present in our DE gene dataset.**

**Supplementary Table S8. All insulin growth factor binding proteins (IGFBPs) and related DE genes identified in our analysis.**

**Supplementary Figure S1.** Venn diagram of coinciding genes between differentially expressed genes in mechanically stressed versus control cartilage from osteochondral explants (DE_MS_) and previously identified differentially expressed genes in preserved versus lesioned OA cartilage (DE_OA_).

**Supplementary Figure S2.** Protein-protein interaction network in STRING of proteins encoded by differentially expressed genes (N=92 genes) not coinciding with OA pathophysiology (DE_ExclusiveMS_).

**Supplementary Figure S3. Heat-map of proteins present in SASP.**

**Supplementary Tables**

**Supplementary Table S1. [a]** Donor characteristics of samples for which RNA was sequenced. [**b**] Donor characteristics of independent samples used for replication of RNA-sequencing findings.
**a**

|  | **Control (N=8 donors)** | **65%MS (N=7 donors)** | **All samples (N=9 donors)** |
| --- | --- | --- | --- |
| **Age (Average ± stdev)** | 61.88 ± 6.06 | 64.29 ± 9.05 | 63.78 ± 8.04 |
| **Age (Range)** | 53-70 | 53-79 | 53-79 |
| **Sex (M/F)** | 3/5 | 3/4 | 3/6 |
| **% Female** | 63% | 57% | 67% |
| **BMI (Average ± stdev)** | 30.36 ± 4.90 | 28.42 ± 3.68 | 29.88 ± 4.80 |
| **BMI (range)** | 24.9-39.2 | 24.9-35.11 | 24.9-39.2 |

**b**

|  | **All samples (N=10 donors)** |
| --- | --- |
| **Age (Average ± stdev)** | 66.90 ± 12.11 |
| **Age (Range)** | 52-85 |
| **Sex (M/F)** | 4/6 |
| **% Female** | 60% |
| **BMI (Average ± stdev)** | 29.09 ± 4.47 |
| **BMI (range)** | 24.78-38.06 |

**Supplementary Table S2. Primer sequences used for replication and validation by RT-qPCR.**

| **Gene name** | **Forward (5'-3')** | **Reverse (5'-3')** |
| --- | --- | --- |
| *SDHA* | TGGAGCTGCAGAACCTGATG | TGTAGTCTTCCCTGGCATGC |
| *YWHAZ* | CTGAGGTTGCAGCTGGTGATGACA | AGCAGGCTTTCTCAGGGGAGTTCA |
| *TNC* | TGTCATCTCCTACACAGGCG | TCGAGGTCGGTCAGAGCATA |
| *IGFBP4* | ATCGAGGCCATCCAGGAAAG | CTGAAGCTGTTGTTGGGGTG |
| *IGFBP5* | GTGCTGTGTACCTGCCCAAT | CGTCAACGTACTCCATGCCT |
| *IGFBP6* | GTCTACCGAGGGGCTCAAAC | GACTTGCCCATCCGATCCAC |
| *CNTFR* | AAGGGCTTCTACTGCAGCTG | CATGTAGCGAATGTGGCAGC |
| *WISP2* | ATGAGAGGCACACCGAAGAC | TGGGTACGCACCTTTGAGAG |
| *FRZB* | ATTGACTTCCAGCACGAGCC | CGAGTGGCGGTACTTGATGAG |
| *COL9A3* | AAGTATCTGCCCGCCAGGTC | TCCCTTGAACCCTGGCATTC |
| *GADD45A* | GCGAGAACGACATCAACATCC | AATGTGGATTCGTCACCAGCA |
| *PTGES* | GGAAGAAGGCCTTTGCCAAC | AGACGAAGCCCAGGAAAAGG |

**Supplementary Table S3. Genes differentially expressed in 65%MS (DE_MS_) cartilage compared to control cartilage of human osteochondral explants.**

| **Ensembl ID** | **Gene Name** | **log2FC** | **FC** | **pvalue** | **FDR** |
| --- | --- | --- | --- | --- | --- |
| ENSG00000167779 | IGFBP6 | -2.37 | 0.19 | 4.72E-08 | 3.07E-04 |
| ENSG00000114126 | TFDP2 | -0.56 | 0.68 | 1.07E-07 | 3.48E-04 |
| ENSG00000100906 | NFKBIA | -0.59 | 0.66 | 2.51E-07 | 5.44E-04 |
| ENSG00000064205 | WISP2 | -1.68 | 0.31 | 9.97E-07 | 1.08E-03 |
| ENSG00000116717 | GADD45A | -0.75 | 0.59 | 6.73E-07 | 1.08E-03 |
| ENSG00000171914 | TLN2 | -1.11 | 0.46 | 8.39E-07 | 1.08E-03 |
| ENSG00000134324 | LPIN1 | -0.87 | 0.55 | 2.79E-06 | 1.79E-03 |
| ENSG00000140105 | WARS | 0.53 | 1.44 | 2.23E-06 | 1.79E-03 |
| ENSG00000157514 | TSC22D3 | -0.77 | 0.58 | 2.66E-06 | 1.79E-03 |
| ENSG00000163453 | IGFBP7 | -1.24 | 0.42 | 2.05E-06 | 1.79E-03 |
| ENSG00000179051 | RCC2 | 0.90 | 1.87 | 3.03E-06 | 1.79E-03 |
| ENSG00000102760 | RGCC | -0.87 | 0.55 | 3.57E-06 | 1.94E-03 |
| ENSG00000196305 | IARS | 0.43 | 1.35 | 4.36E-06 | 2.18E-03 |
| ENSG00000144908 | ALDH1L1 | -1.62 | 0.33 | 4.78E-06 | 2.22E-03 |
| ENSG00000068383 | INPP5A | -0.77 | 0.58 | 5.60E-06 | 2.43E-03 |
| ENSG00000126803 | HSPA2 | -0.85 | 0.55 | 7.74E-06 | 2.65E-03 |
| ENSG00000143416 | SELENBP1 | -1.09 | 0.47 | 7.67E-06 | 2.65E-03 |
| ENSG00000148498 | PARD3 | -0.78 | 0.58 | 6.56E-06 | 2.65E-03 |
| ENSG00000159461 | AMFR | -0.54 | 0.69 | 6.98E-06 | 2.65E-03 |
| ENSG00000204103 | MAFB | 1.57 | 2.97 | 1.43E-05 | 4.66E-03 |
| ENSG00000119408 | NEK6 | 1.18 | 2.26 | 1.91E-05 | 5.86E-03 |
| ENSG00000142871 | CYR61 | -0.65 | 0.64 | 1.98E-05 | 5.86E-03 |
| ENSG00000138356 | AOX1 | -1.06 | 0.48 | 2.18E-05 | 6.10E-03 |
| ENSG00000155324 | GRAMD2B | -0.80 | 0.58 | 2.25E-05 | 6.10E-03 |
| ENSG00000107736 | CDH23 | -1.44 | 0.37 | 2.72E-05 | 6.88E-03 |
| ENSG00000150907 | FOXO1 | -0.68 | 0.62 | 2.86E-05 | 6.88E-03 |
| ENSG00000163686 | ABHD6 | -1.05 | 0.48 | 2.96E-05 | 6.88E-03 |
| ENSG00000167191 | GPRC5B | -0.87 | 0.55 | 2.94E-05 | 6.88E-03 |
| ENSG00000100612 | DHRS7 | -0.48 | 0.71 | 3.97E-05 | 7.81E-03 |
| ENSG00000115461 | IGFBP5 | 2.59 | 6.01 | 3.54E-05 | 7.81E-03 |
| ENSG00000115468 | EFHD1 | -0.80 | 0.57 | 3.73E-05 | 7.81E-03 |
| ENSG00000134107 | BHLHE40 | 1.00 | 2.00 | 4.08E-05 | 7.81E-03 |
| ENSG00000146122 | DAAM2 | -0.95 | 0.52 | 4.08E-05 | 7.81E-03 |
| ENSG00000173641 | HSPB7 | -0.89 | 0.54 | 3.81E-05 | 7.81E-03 |
| ENSG00000135069 | PSAT1 | 1.03 | 2.04 | 4.52E-05 | 8.29E-03 |
| ENSG00000148344 | PTGES | 1.55 | 2.92 | 4.58E-05 | 8.29E-03 |
| ENSG00000166348 | USP54 | -0.76 | 0.59 | 4.79E-05 | 8.43E-03 |
| ENSG00000139514 | SLC7A1 | 0.57 | 1.48 | 4.93E-05 | 8.44E-03 |
| ENSG00000041982 | TNC | 1.48 | 2.80 | 5.15E-05 | 8.51E-03 |
| ENSG00000162998 | FRZB | -1.63 | 0.32 | 5.23E-05 | 8.51E-03 |
| ENSG00000132970 | WASF3 | -0.41 | 0.75 | 5.36E-05 | 8.51E-03 |
| ENSG00000103257 | SLC7A5 | 0.89 | 1.86 | 6.44E-05 | 9.96E-03 |
| ENSG00000104324 | CPQ | -0.46 | 0.72 | 6.58E-05 | 9.96E-03 |
| ENSG00000166165 | CKB | -1.39 | 0.38 | 7.19E-05 | 1.04E-02 |
| ENSG00000168918 | INPP5D | -1.25 | 0.42 | 7.13E-05 | 1.04E-02 |
| ENSG00000129757 | CDKN1C | -1.23 | 0.43 | 7.90E-05 | 1.09E-02 |
| ENSG00000143878 | RHOB | -0.61 | 0.66 | 8.05E-05 | 1.09E-02 |
| ENSG00000183864 | TOB2 | -0.71 | 0.61 | 7.96E-05 | 1.09E-02 |
| ENSG00000053747 | LAMA3 | -1.15 | 0.45 | 9.40E-05 | 1.25E-02 |
| ENSG00000145246 | ATP10D | -0.49 | 0.71 | 9.96E-05 | 1.30E-02 |
| ENSG00000101825 | MXRA5 | 1.21 | 2.32 | 1.11E-04 | 1.42E-02 |
| ENSG00000122756 | CNTFR | -1.88 | 0.27 | 1.15E-04 | 1.44E-02 |
| ENSG00000117020 | AKT3 | -0.44 | 0.74 | 1.26E-04 | 1.44E-02 |
| ENSG00000134954 | ETS1 | 0.46 | 1.38 | 1.26E-04 | 1.44E-02 |
| ENSG00000165966 | PDZRN4 | -1.97 | 0.26 | 1.26E-04 | 1.44E-02 |
| ENSG00000169116 | PARM1 | -1.08 | 0.47 | 1.26E-04 | 1.44E-02 |
| ENSG00000187720 | THSD4 | -1.53 | 0.35 | 1.18E-04 | 1.44E-02 |
| ENSG00000113739 | STC2 | -1.07 | 0.48 | 1.39E-04 | 1.53E-02 |
| ENSG00000180354 | MTURN | -0.60 | 0.66 | 1.37E-04 | 1.53E-02 |
| ENSG00000185630 | PBX1 | -0.70 | 0.62 | 1.41E-04 | 1.53E-02 |
| ENSG00000117151 | CTBS | -0.44 | 0.73 | 1.53E-04 | 1.63E-02 |
| ENSG00000136997 | MYC | 0.79 | 1.73 | 1.56E-04 | 1.64E-02 |
| ENSG00000080546 | SESN1 | -0.84 | 0.56 | 1.78E-04 | 1.84E-02 |
| ENSG00000198755 | RPL10A | 0.34 | 1.26 | 1.88E-04 | 1.91E-02 |
| ENSG00000080298 | RFX3 | -0.58 | 0.67 | 1.99E-04 | 1.99E-02 |
| ENSG00000114166 | KAT2B | -0.65 | 0.64 | 2.09E-04 | 2.06E-02 |
| ENSG00000127528 | KLF2 | -1.18 | 0.44 | 2.15E-04 | 2.09E-02 |
| ENSG00000106991 | ENG | 0.59 | 1.50 | 2.31E-04 | 2.21E-02 |
| ENSG00000100242 | SUN2 | -0.82 | 0.57 | 2.39E-04 | 2.25E-02 |
| ENSG00000100029 | PES1 | 0.44 | 1.36 | 2.49E-04 | 2.28E-02 |
| ENSG00000185201 | IFITM2 | 0.72 | 1.65 | 2.46E-04 | 2.28E-02 |
| ENSG00000111640 | GAPDH | 0.58 | 1.49 | 2.53E-04 | 2.29E-02 |
| ENSG00000173848 | NET1 | -0.90 | 0.54 | 2.65E-04 | 2.36E-02 |
| ENSG00000138336 | TET1 | -0.47 | 0.72 | 2.75E-04 | 2.42E-02 |
| ENSG00000101782 | RIOK3 | -0.44 | 0.74 | 2.81E-04 | 2.43E-02 |
| ENSG00000159322 | ADPGK | 0.35 | 1.27 | 2.83E-04 | 2.43E-02 |
| ENSG00000111885 | MAN1A1 | -0.99 | 0.50 | 2.94E-04 | 2.48E-02 |
| ENSG00000089220 | PEBP1 | -0.62 | 0.65 | 3.04E-04 | 2.54E-02 |
| ENSG00000005022 | SLC25A5 | 0.48 | 1.40 | 3.16E-04 | 2.56E-02 |
| ENSG00000137699 | TRIM29 | -1.13 | 0.46 | 3.18E-04 | 2.56E-02 |
| ENSG00000166033 | HTRA1 | 0.95 | 1.93 | 3.17E-04 | 2.56E-02 |
| ENSG00000184205 | TSPYL2 | -0.75 | 0.60 | 3.42E-04 | 2.71E-02 |
| ENSG00000143382 | ADAMTSL4 | -0.66 | 0.63 | 3.58E-04 | 2.81E-02 |
| ENSG00000062716 | VMP1 | 0.60 | 1.52 | 3.89E-04 | 3.01E-02 |
| ENSG00000067141 | NEO1 | -0.80 | 0.58 | 4.24E-04 | 3.10E-02 |
| ENSG00000078596 | ITM2A | -1.00 | 0.50 | 4.18E-04 | 3.10E-02 |
| ENSG00000096060 | FKBP5 | -0.64 | 0.64 | 4.07E-04 | 3.10E-02 |
| ENSG00000134109 | EDEM1 | 0.33 | 1.26 | 4.20E-04 | 3.10E-02 |
| ENSG00000148180 | GSN | -0.60 | 0.66 | 4.16E-04 | 3.10E-02 |
| ENSG00000157600 | TMEM164 | -0.73 | 0.60 | 4.28E-04 | 3.10E-02 |
| ENSG00000143164 | DCAF6 | -0.22 | 0.86 | 4.34E-04 | 3.10E-02 |
| ENSG00000071205 | ARHGAP10 | -0.47 | 0.72 | 4.57E-04 | 3.14E-02 |
| ENSG00000079691 | CARMIL1 | -0.55 | 0.68 | 4.51E-04 | 3.14E-02 |
| ENSG00000131844 | MCCC2 | -0.89 | 0.54 | 4.58E-04 | 3.14E-02 |
| ENSG00000168874 | ATOH8 | -0.91 | 0.53 | 4.53E-04 | 3.14E-02 |
| ENSG00000106624 | AEBP1 | 0.79 | 1.73 | 4.98E-04 | 3.31E-02 |
| ENSG00000154380 | ENAH | 0.53 | 1.44 | 4.97E-04 | 3.31E-02 |
| ENSG00000253293 | HOXA10 | -0.36 | 0.78 | 4.97E-04 | 3.31E-02 |
| ENSG00000006016 | CRLF1 | 0.76 | 1.69 | 5.15E-04 | 3.38E-02 |
| ENSG00000106366 | SERPINE1 | 1.02 | 2.03 | 5.33E-04 | 3.47E-02 |
| ENSG00000141753 | IGFBP4 | 1.38 | 2.59 | 5.44E-04 | 3.50E-02 |
| ENSG00000179532 | DNHD1 | -0.51 | 0.70 | 5.53E-04 | 3.53E-02 |
| ENSG00000106258 | CYP3A5 | -1.40 | 0.38 | 5.74E-04 | 3.62E-02 |
| ENSG00000082196 | C1QTNF3 | -1.73 | 0.30 | 5.86E-04 | 3.64E-02 |
| ENSG00000164106 | SCRG1 | -0.89 | 0.54 | 5.87E-04 | 3.64E-02 |
| ENSG00000151332 | MBIP | -0.40 | 0.76 | 6.10E-04 | 3.71E-02 |
| ENSG00000187840 | EIF4EBP1 | 1.06 | 2.09 | 6.04E-04 | 3.71E-02 |
| ENSG00000132824 | SERINC3 | -0.32 | 0.80 | 6.54E-04 | 3.91E-02 |
| ENSG00000135506 | OS9 | -0.33 | 0.80 | 6.48E-04 | 3.91E-02 |
| ENSG00000148848 | ADAM12 | 0.62 | 1.54 | 6.63E-04 | 3.92E-02 |
| ENSG00000013016 | EHD3 | 0.42 | 1.34 | 6.75E-04 | 3.93E-02 |
| ENSG00000101439 | CST3 | -0.74 | 0.60 | 6.77E-04 | 3.93E-02 |
| ENSG00000179941 | BBS10 | -0.57 | 0.67 | 6.83E-04 | 3.93E-02 |
| ENSG00000104714 | ERICH1 | -0.46 | 0.73 | 7.07E-04 | 3.93E-02 |
| ENSG00000106351 | AGFG2 | -0.68 | 0.63 | 6.97E-04 | 3.93E-02 |
| ENSG00000158825 | CDA | -1.07 | 0.48 | 7.07E-04 | 3.93E-02 |
| ENSG00000184557 | SOCS3 | 0.78 | 1.71 | 7.07E-04 | 3.93E-02 |
| ENSG00000162627 | SNX7 | -0.56 | 0.68 | 7.13E-04 | 3.93E-02 |
| ENSG00000136295 | TTYH3 | 1.05 | 2.08 | 7.27E-04 | 3.97E-02 |
| ENSG00000092445 | TYRO3 | 0.77 | 1.71 | 7.45E-04 | 4.04E-02 |
| ENSG00000010278 | CD9 | -0.80 | 0.57 | 7.82E-04 | 4.11E-02 |
| ENSG00000049860 | HEXB | -0.46 | 0.73 | 7.79E-04 | 4.11E-02 |
| ENSG00000165795 | NDRG2 | -0.72 | 0.61 | 7.65E-04 | 4.11E-02 |
| ENSG00000170891 | CYTL1 | -1.65 | 0.32 | 7.72E-04 | 4.11E-02 |
| ENSG00000187514 | PTMA | 0.49 | 1.40 | 8.00E-04 | 4.17E-02 |
| ENSG00000112559 | MDFI | 0.76 | 1.69 | 8.45E-04 | 4.36E-02 |
| ENSG00000159307 | SCUBE1 | -0.93 | 0.53 | 8.50E-04 | 4.36E-02 |
| ENSG00000144857 | BOC | -1.08 | 0.47 | 8.75E-04 | 4.45E-02 |
| ENSG00000131067 | GGT7 | -0.54 | 0.69 | 8.90E-04 | 4.45E-02 |
| ENSG00000171150 | SOCS5 | -0.40 | 0.76 | 8.85E-04 | 4.45E-02 |
| ENSG00000112306 | RPS12 | 0.26 | 1.19 | 9.15E-04 | 4.54E-02 |
| ENSG00000119938 | PPP1R3C | -0.71 | 0.61 | 9.54E-04 | 4.68E-02 |
| ENSG00000164237 | CMBL | -0.57 | 0.67 | 9.56E-04 | 4.68E-02 |
| ENSG00000090530 | P3H2 | -1.17 | 0.44 | 1.02E-03 | 4.71E-02 |
| ENSG00000106617 | PRKAG2 | -0.51 | 0.70 | 1.01E-03 | 4.71E-02 |
| ENSG00000112118 | MCM3 | 0.39 | 1.31 | 1.01E-03 | 4.71E-02 |
| ENSG00000117868 | ESYT2 | -0.18 | 0.88 | 9.79E-04 | 4.71E-02 |
| ENSG00000141258 | SGSM2 | -0.87 | 0.55 | 1.01E-03 | 4.71E-02 |
| ENSG00000143127 | ITGA10 | -0.47 | 0.72 | 9.98E-04 | 4.71E-02 |
| ENSG00000149485 | FADS1 | -0.65 | 0.64 | 1.04E-03 | 4.71E-02 |
| ENSG00000162804 | SNED1 | 0.87 | 1.83 | 1.04E-03 | 4.71E-02 |
| ENSG00000169184 | MN1 | -0.80 | 0.58 | 1.04E-03 | 4.71E-02 |
| ENSG00000169902 | TPST1 | 0.36 | 1.28 | 1.04E-03 | 4.71E-02 |
| ENSG00000185634 | SHC4 | 1.05 | 2.07 | 9.91E-04 | 4.71E-02 |
| ENSG00000130635 | COL5A1 | 0.96 | 1.95 | 1.06E-03 | 4.78E-02 |
| ENSG00000004799 | PDK4 | -0.88 | 0.54 | 1.13E-03 | 4.84E-02 |
| ENSG00000105974 | CAV1 | -0.51 | 0.70 | 1.12E-03 | 4.84E-02 |
| ENSG00000131389 | SLC6A6 | 0.98 | 1.97 | 1.12E-03 | 4.84E-02 |
| ENSG00000137745 | MMP13 | 2.38 | 5.19 | 1.13E-03 | 4.84E-02 |
| ENSG00000172348 | RCAN2 | -0.88 | 0.54 | 1.10E-03 | 4.84E-02 |
| ENSG00000172493 | AFF1 | -0.39 | 0.76 | 1.09E-03 | 4.84E-02 |
| ENSG00000187151 | ANGPTL5 | -1.18 | 0.44 | 1.11E-03 | 4.84E-02 |
| ENSG00000100814 | CCNB1IP1 | -0.33 | 0.80 | 1.14E-03 | 4.84E-02 |
| ENSG00000092758 | COL9A3 | -1.62 | 0.32 | 1.16E-03 | 4.89E-02 |
| ENSG00000160111 | CPAMD8 | -1.37 | 0.39 | 1.19E-03 | 4.98E-02 |
| ENSG00000170776 | AKAP13 | -0.41 | 0.75 | 1.19E-03 | 4.98E-02 |

Legend: Log2FC, log2 fold change; FC, fold change; FDR, False discovery rate

**Supplementary Table S4. Gene enrichment found in Enrichr.** Enrichment for **[a]** 156 DE_MS_ genes in and **[b]** 92 DE_ExclusiveMS_ for the gene ontology terms: biological process, molecular function and cellular component 2018, and pathways: KEGG 2019 human and reactome.

**a**

| **Term** | **Overlap** | **P-value** | **Adj P-value** | **Odds Ratio** | **Combined Score** | **Genes** |
| --- | --- | --- | --- | --- | --- | --- |
| endoplasmic reticulum lumen (GO:0005788) | 12/270 | 1.44E-06 | 6.42E-04 | 5.70 | 76.64 | CST3;IGFBP5;COL5A1;OS9;IGFBP4;ADAMTSL4;STC2;TNC;P3H2;IGFBP7;COL9A3;CYR61 |
| negative regulation of cellular process (GO:0048523) | 17/534 | 9.82E-07 | 5.01E-03 | 4.08 | 56.46 | TSPYL2;CDA;WARS;CAV1;P3H2;ETS1;CYR61;WISP2;RHOB;KAT2B;RGCC;FRZB;MYC;BHLHE40;CD9;IGFBP7;IGFBP6 |
| Cellular senescence | 8/160 | 3.75E-05 | 1.15E-02 | 6.41 | 65.33 | GADD45A;MYC;SERPINE1;AKT3;EIF4EBP1;SLC25A5;ETS1;FOXO1 |
| protein kinase regulator activity (GO:0019887) | 7/107 | 2.08E-05 | 1.20E-02 | 8.39 | 90.40 | CDKN1C;SOCS3;GPRC5B;RGCC;MBIP;PRKAG2;SOCS5 |
| Insulin resistance | 6/108 | 2.03E-04 | 1.25E-02 | 7.12 | 60.55 | NFKBIA;SOCS3;PPP1R3C;AKT3;PRKAG2;FOXO1 |
| Focal adhesion | 8/199 | 1.72E-04 | 1.33E-02 | 5.15 | 44.67 | SHC4;CAV1;ITGA10;AKT3;LAMA3;TNC;COL9A3;TLN2 |
| Longevity regulating pathway | 6/102 | 1.49E-04 | 1.53E-02 | 7.54 | 66.47 | SESN1;AKT3;EIF4EBP1;PRKAG2;HSPA2;FOXO1 |
| Insulin signaling pathway | 7/137 | 1.01E-04 | 1.56E-02 | 6.55 | 60.26 | SHC4;SOCS3;PPP1R3C;AKT3;EIF4EBP1;PRKAG2;FOXO1 |
| Chronic myeloid leukemia | 5/76 | 3.20E-04 | 1.64E-02 | 8.43 | 67.87 | SHC4;NFKBIA;GADD45A;MYC;AKT3 |
| insulin-like growth factor II binding (GO:0031995) | 3/7 | 1.59E-05 | 1.83E-02 | 54.95 | 607.02 | IGFBP5;IGFBP4;IGFBP6 |
| focal adhesion (GO:0005925) | 11/356 | 1.14E-04 | 2.54E-02 | 3.96 | 35.97 | ENAH;EHD3;GSN;CAV1;TNC;CD9;TLN2;RPL10A;DCAF6;RHOB;ENG |
| insulin-like growth factor I binding (GO:0031994) | 3/13 | 1.26E-04 | 2.89E-02 | 29.59 | 265.73 | IGFBP5;IGFBP4;IGFBP6 |
| insulin-like growth factor binding (GO:0005520) | 3/14 | 1.59E-04 | 3.05E-02 | 27.47 | 240.28 | IGFBP5;IGFBP4;IGFBP6 |
| kinase binding (GO:0019900) | 12/418 | 1.11E-04 | 3.20E-02 | 3.68 | 33.51 | SHC4;KAT2B;GPRC5B;RGCC;WARS;GADD45A;CAV1;RCC2;NEK6;PEBP1;PRKAG2;RHOB |
| extracellular matrix organization (GO:0030198) | 10/229 | 1.30E-05 | 3.31E-02 | 5.60 | 62.99 | SCUBE1;MMP13;COL5A1;ITGA10;SERPINE1;ADAM12;LAMA3;TNC;HTRA1;COL9A3 |
| Vitamin B6 metabolism | 2/6 | 8.88E-04 | 3.42E-02 | 42.74 | 300.26 | PSAT1;AOX1 |
| Small cell lung cancer | 5/93 | 8.10E-04 | 3.56E-02 | 6.89 | 49.06 | NFKBIA;GADD45A;MYC;AKT3;LAMA3 |
| kinase inhibitor activity (GO:0019210) | 5/59 | 9.65E-05 | 3.70E-02 | 10.86 | 100.45 | CDKN1C;SOCS3;WARS;MBIP;SOCS5 |
| protein alpha-1,2-demannosylation (GO:0036508) | 4/24 | 3.35E-05 | 4.27E-02 | 21.37 | 220.16 | OS9;EDEM1;AMFR;MAN1A1 |
| negative regulation of cell proliferation (GO:0008285) | 12/363 | 2.88E-05 | 4.90E-02 | 4.24 | 44.31 | CDKN1C;KAT2B;RGCC;WARS;IGFBP5;FRZB;MYC;BHLHE40;P3H2;IGFBP7;IGFBP6;ETS1 |

**b**

| **Term** | **Overlap** | **P-value** | **Adj P-value** | **Odds Ratio** | **Combined Score** | **Genes** |
| --- | --- | --- | --- | --- | --- | --- |
| kinase inhibitor activity (GO:0019210) | 5/59 | 7.59E-06 | 8.74E-03 | 18.42 | 217.17 | CDKN1C;SOCS3;WARS;MBIP ;SOCS5 |
| focal adhesion (GO:0005925) | 9/356 | 3.84E-05 | 1.71E-02 | 5.50 | 55.88 | ENAH;EHD3;GSN;CD9;TLN2;RPL10A;DCAF6;RHOB;ENG |

**Supplementary Table S5. DE_MS_ genes coinciding with previously reported DE genes in OA pathophysiology (DE_OA_). [a]** DE_MS_ genes with same direction of effect as DE_OA_ genes. **[b]** DE_MS_ genes with opposite direction of effect as DE_OA_ genes.

| **a** | **65%MS versus control cartilage (n=14/group)** | | | | **Lesioned versus Preserved OA cartilage (n=35/group)** | | | |
| --- | --- | --- | --- | --- | --- | --- | --- | --- |
| **Gene Name** | **log2FC** | **FC** | **pvalue** | **FDR** | **log2FC** | **FC** | **pvalue** | **FDR** |
| *TFDP2* | -0.56 | 0.68 | 1.07E-07 | 3.48E-04 | -0.23 | 0.85 | 2.02E-03 | 2.27E-02 |
| *WISP2* | -1.68 | 0.31 | 9.97E-07 | 1.08E-03 | -1.20 | 0.44 | 1.48E-04 | 3.06E-03 |
| *ALDH1L1* | -1.62 | 0.33 | 4.78E-06 | 2.22E-03 | -1.02 | 0.49 | 5.56E-09 | 7.24E-07 |
| *INPP5A* | -0.77 | 0.58 | 5.60E-06 | 2.43E-03 | -0.31 | 0.81 | 1.69E-03 | 1.99E-02 |
| *GRAMD2B* | -0.80 | 0.58 | 2.25E-05 | 6.10E-03 | -0.30 | 0.81 | 1.18E-04 | 2.54E-03 |
| *GPRC5B* | -0.87 | 0.55 | 2.94E-05 | 6.88E-03 | -1.02 | 0.49 | 2.10E-09 | 3.32E-07 |
| *DAAM2* | -0.95 | 0.52 | 4.08E-05 | 7.81E-03 | -0.50 | 0.71 | 2.40E-04 | 4.48E-03 |
| *EFHD1* | -0.80 | 0.57 | 3.73E-05 | 7.81E-03 | -0.40 | 0.76 | 2.37E-03 | 2.54E-02 |
| *FRZB* | -1.63 | 0.32 | 5.23E-05 | 8.51E-03 | -1.88 | 0.27 | 4.07E-12 | 1.87E-09 |
| *TOB2* | -0.71 | 0.61 | 7.96E-05 | 1.09E-02 | -0.26 | 0.84 | 4.72E-05 | 1.22E-03 |
| *CNTFR* | -1.88 | 0.27 | 1.15E-04 | 1.44E-02 | -1.69 | 0.31 | 3.32E-12 | 1.56E-09 |
| *PDZRN4* | -1.97 | 0.26 | 1.26E-04 | 1.44E-02 | -1.37 | 0.39 | 2.70E-05 | 7.76E-04 |
| *PARM1* | -1.08 | 0.47 | 1.26E-04 | 1.44E-02 | -0.65 | 0.64 | 2.63E-05 | 7.61E-04 |
| *STC2* | -1.07 | 0.48 | 1.39E-04 | 1.53E-02 | -1.03 | 0.49 | 1.05E-08 | 1.24E-06 |
| *MTURN* | -0.60 | 0.66 | 1.37E-04 | 1.53E-02 | -0.42 | 0.75 | 3.76E-06 | 1.58E-04 |
| *KAT2B* | -0.65 | 0.64 | 2.09E-04 | 2.06E-02 | -0.33 | 0.80 | 1.33E-03 | 1.66E-02 |
| *ADAMTSL4* | -0.66 | 0.63 | 3.58E-04 | 2.81E-02 | -0.42 | 0.75 | 1.26E-03 | 1.61E-02 |
| *ITM2A* | -1.00 | 0.50 | 4.18E-04 | 3.10E-02 | -0.60 | 0.66 | 8.86E-05 | 2.02E-03 |
| *NEO1* | -0.80 | 0.58 | 4.24E-04 | 3.10E-02 | -0.32 | 0.80 | 1.38E-03 | 1.71E-02 |
| *FKBP5* | -0.64 | 0.64 | 4.07E-04 | 3.10E-02 | -0.20 | 0.87 | 5.39E-03 | 4.59E-02 |
| *ATOH8* | -0.91 | 0.53 | 4.53E-04 | 3.14E-02 | -0.48 | 0.72 | 1.67E-03 | 1.97E-02 |
| *HOXA10* | -0.36 | 0.78 | 4.97E-04 | 3.31E-02 | -0.36 | 0.78 | 2.32E-04 | 4.37E-03 |
| *AGFG2* | -0.68 | 0.63 | 6.97E-04 | 3.93E-02 | -0.30 | 0.81 | 5.47E-03 | 4.64E-02 |
| *NDRG2* | -0.72 | 0.61 | 7.65E-04 | 4.11E-02 | -0.70 | 0.62 | 1.67E-08 | 1.83E-06 |
| *CYTL1* | -1.65 | 0.32 | 7.72E-04 | 4.11E-02 | -0.46 | 0.73 | 4.94E-03 | 4.31E-02 |
| *SCUBE1* | -0.93 | 0.53 | 8.50E-04 | 4.36E-02 | -1.26 | 0.42 | 2.03E-08 | 2.15E-06 |
| *BOC* | -1.08 | 0.47 | 8.75E-04 | 4.45E-02 | -0.70 | 0.62 | 3.96E-07 | 2.54E-05 |
| *CMBL* | -0.57 | 0.67 | 9.56E-04 | 4.68E-02 | -0.41 | 0.75 | 1.34E-07 | 1.02E-05 |
| *RCAN2* | -0.88 | 0.54 | 1.10E-03 | 4.84E-02 | -2.03 | 0.25 | 8.20E-15 | 9.19E-12 |
| *CPAMD8* | -1.37 | 0.39 | 1.19E-03 | 4.98E-02 | -1.19 | 0.44 | 2.90E-09 | 4.23E-07 |
| *IARS* | 0.43 | 1.35 | 4.36E-06 | 2.18E-03 | 0.40 | 1.32 | 1.35E-04 | 2.85E-03 |
| *BHLHE40* | 1.00 | 2.00 | 4.08E-05 | 7.81E-03 | 0.30 | 1.23 | 4.89E-04 | 7.78E-03 |
| *PSAT1* | 1.03 | 2.04 | 4.52E-05 | 8.29E-03 | 0.77 | 1.70 | 1.14E-03 | 1.49E-02 |
| *PTGES* | 1.55 | 2.92 | 4.58E-05 | 8.29E-03 | 1.61 | 3.06 | 2.33E-15 | 3.61E-12 |
| *SLC7A1* | 0.57 | 1.48 | 4.93E-05 | 8.44E-03 | 0.53 | 1.44 | 1.43E-06 | 7.10E-05 |
| *TNC* | 1.48 | 2.80 | 5.15E-05 | 8.51E-03 | 0.50 | 1.41 | 7.53E-04 | 1.09E-02 |
| *SLC7A5* | 0.89 | 1.86 | 6.44E-05 | 9.96E-03 | 0.99 | 1.99 | 2.91E-10 | 6.25E-08 |
| *MXRA5* | 1.21 | 2.32 | 1.11E-04 | 1.42E-02 | 0.53 | 1.44 | 3.42E-04 | 5.94E-03 |
| *HTRA1* | 0.95 | 1.93 | 3.17E-04 | 2.56E-02 | 1.26 | 2.39 | 1.80E-14 | 1.65E-11 |
| *EDEM1* | 0.33 | 1.26 | 4.20E-04 | 3.10E-02 | 0.23 | 1.17 | 2.38E-03 | 2.55E-02 |
| *CRLF1* | 0.76 | 1.69 | 5.15E-04 | 3.38E-02 | 1.60 | 3.04 | 4.59E-13 | 2.96E-10 |
| *SERPINE1* | 1.02 | 2.03 | 5.33E-04 | 3.47E-02 | 1.57 | 2.97 | 3.22E-13 | 2.24E-10 |
| *IGFBP4* | 1.38 | 2.59 | 5.44E-04 | 3.50E-02 | 0.67 | 1.60 | 2.27E-03 | 2.47E-02 |
| *ADAM12* | 0.62 | 1.54 | 6.63E-04 | 3.92E-02 | 0.99 | 1.98 | 4.58E-06 | 1.85E-04 |
| *TYRO3* | 0.77 | 1.71 | 7.45E-04 | 4.04E-02 | 0.94 | 1.92 | 1.19E-11 | 5.00E-09 |
| *SHC4* | 1.05 | 2.07 | 9.91E-04 | 4.71E-02 | 1.07 | 2.10 | 1.43E-11 | 5.87E-09 |
| *COL5A1* | 0.96 | 1.95 | 1.06E-03 | 4.78E-02 | 0.46 | 1.38 | 4.94E-03 | 4.31E-02 |
| *SLC6A6* | 0.98 | 1.97 | 1.12E-03 | 4.84E-02 | 0.60 | 1.51 | 1.13E-04 | 2.45E-03 |

Legend: Log2FC, log2 fold change; FC, fold change; FDR, False discovery rate

| **b** | **Control versus 65% MS cartilage (n=14/group)** | | | | **Lesioned versus Preserved OA cartilage (n=35/group)** | | | |
| --- | --- | --- | --- | --- | --- | --- | --- | --- |
| **Gene Name** | **log2FC** | **FC** | **pvalue** | **FDR** | **log2FC** | **FC** | **pvalue** | **FDR** |
| *TMEM164* | -0.73 | 0.60 | 4.28E-04 | 3.10E-02 | 0.24 | 1.18 | 5.09E-03 | 4.41E-02 |
| *FOXO1* | -0.68 | 0.62 | 2.86E-05 | 6.88E-03 | 0.29 | 1.22 | 6.52E-04 | 9.72E-03 |
| *CAV1* | -0.51 | 0.70 | 1.12E-03 | 4.84E-02 | 0.33 | 1.25 | 4.58E-03 | 4.07E-02 |
| *AKAP13* | -0.41 | 0.75 | 1.19E-03 | 4.98E-02 | 0.33 | 1.26 | 1.11E-04 | 2.42E-03 |
| *CDA* | -1.07 | 0.48 | 7.07E-04 | 3.93E-02 | 0.39 | 1.31 | 2.31E-03 | 2.50E-02 |
| *IGFBP7* | -1.24 | 0.42 | 2.05E-06 | 1.79E-03 | 0.47 | 1.38 | 3.42E-03 | 3.30E-02 |
| *RGCC* | -0.87 | 0.55 | 3.57E-06 | 1.94E-03 | 0.47 | 1.39 | 1.59E-05 | 5.13E-04 |
| *AKT3* | -0.44 | 0.74 | 1.26E-04 | 1.44E-02 | 0.52 | 1.43 | 1.01E-04 | 2.25E-03 |
| *SNX7* | -0.56 | 0.68 | 7.13E-04 | 3.93E-02 | 0.53 | 1.45 | 3.24E-04 | 5.67E-03 |
| *C1QTNF3* | -1.73 | 0.30 | 5.86E-04 | 3.64E-02 | 0.60 | 1.52 | 9.93E-04 | 1.35E-02 |
| *CKB* | -1.39 | 0.38 | 7.19E-05 | 1.04E-02 | 0.63 | 1.55 | 1.37E-03 | 1.70E-02 |
| *TSC22D3* | -0.77 | 0.58 | 2.66E-06 | 1.79E-03 | 0.67 | 1.59 | 2.81E-09 | 4.16E-07 |
| *TRIM29* | -1.13 | 0.46 | 3.18E-04 | 2.56E-02 | 0.78 | 1.72 | 1.48E-05 | 4.85E-04 |
| *P3H2* | -1.17 | 0.44 | 1.02E-03 | 4.71E-02 | 1.69 | 3.23 | 0.00E+00 | 0.00E+00 |
| *MAFB* | 1.57 | 2.97 | 1.43E-05 | 4.66E-03 | -0.69 | 0.62 | 1.32E-03 | 1.66E-02 |
| *MDFI* | 0.76 | 1.69 | 8.45E-04 | 4.36E-02 | -0.42 | 0.75 | 5.76E-03 | 4.82E-02 |

Legend: Log2FC, log2 fold change; FC, fold change; FDR, False discovery rate

**Supplementary Table S6. Exclusive mechanical response genes (DE_ExclusiveMS_).**

| **Gene name** | **log2FC** | **FC** | **pvalue** | **FDR** |
| --- | --- | --- | --- | --- |
| IGFBP6 | -2.37 | 0.19 | 4.72E-08 | 3.07E-04 |
| NFKBIA | -0.59 | 0.66 | 2.51E-07 | 5.44E-04 |
| TLN2 | -1.11 | 0.46 | 8.39E-07 | 1.08E-03 |
| GADD45A | -0.75 | 0.59 | 6.73E-07 | 1.08E-03 |
| RCC2 | 0.90 | 1.87 | 3.03E-06 | 1.79E-03 |
| WARS | 0.53 | 1.44 | 2.23E-06 | 1.79E-03 |
| LPIN1 | -0.87 | 0.55 | 2.79E-06 | 1.79E-03 |
| AMFR | -0.54 | 0.69 | 6.98E-06 | 2.65E-03 |
| PARD3 | -0.78 | 0.58 | 6.56E-06 | 2.65E-03 |
| SELENBP1 | -1.09 | 0.47 | 7.67E-06 | 2.65E-03 |
| HSPA2 | -0.85 | 0.55 | 7.74E-06 | 2.65E-03 |
| CYR61 | -0.65 | 0.64 | 1.98E-05 | 5.86E-03 |
| NEK6 | 1.18 | 2.26 | 1.91E-05 | 5.86E-03 |
| AOX1 | -1.06 | 0.48 | 2.18E-05 | 6.10E-03 |
| ABHD6 | -1.05 | 0.48 | 2.96E-05 | 6.88E-03 |
| CDH23 | -1.44 | 0.37 | 2.72E-05 | 6.88E-03 |
| HSPB7 | -0.89 | 0.54 | 3.81E-05 | 7.81E-03 |
| IGFBP5 | 2.59 | 6.01 | 3.54E-05 | 7.81E-03 |
| DHRS7 | -0.48 | 0.71 | 3.97E-05 | 7.81E-03 |
| USP54 | -0.76 | 0.59 | 4.79E-05 | 8.43E-03 |
| WASF3 | -0.41 | 0.75 | 5.36E-05 | 8.51E-03 |
| CPQ | -0.46 | 0.72 | 6.58E-05 | 9.96E-03 |
| INPP5D | -1.25 | 0.42 | 7.13E-05 | 1.04E-02 |
| RHOB | -0.61 | 0.66 | 8.05E-05 | 1.09E-02 |
| CDKN1C | -1.23 | 0.43 | 7.90E-05 | 1.09E-02 |
| LAMA3 | -1.15 | 0.45 | 9.40E-05 | 1.25E-02 |
| ATP10D | -0.49 | 0.71 | 9.96E-05 | 1.30E-02 |
| THSD4 | -1.53 | 0.35 | 1.18E-04 | 1.44E-02 |
| ETS1 | 0.46 | 1.38 | 1.26E-04 | 1.44E-02 |
| PBX1 | -0.70 | 0.62 | 1.41E-04 | 1.53E-02 |
| CTBS | -0.44 | 0.73 | 1.53E-04 | 1.63E-02 |
| MYC | 0.79 | 1.73 | 1.56E-04 | 1.64E-02 |
| SESN1 | -0.84 | 0.56 | 1.78E-04 | 1.84E-02 |
| RPL10A | 0.34 | 1.26 | 1.88E-04 | 1.91E-02 |
| RFX3 | -0.58 | 0.67 | 1.99E-04 | 1.99E-02 |
| KLF2 | -1.18 | 0.44 | 2.15E-04 | 2.09E-02 |
| ENG | 0.59 | 1.50 | 2.31E-04 | 2.21E-02 |
| SUN2 | -0.82 | 0.57 | 2.39E-04 | 2.25E-02 |
| IFITM2 | 0.72 | 1.65 | 2.46E-04 | 2.28E-02 |
| PES1 | 0.44 | 1.36 | 2.49E-04 | 2.28E-02 |
| GAPDH | 0.58 | 1.49 | 2.53E-04 | 2.29E-02 |
| NET1 | -0.90 | 0.54 | 2.65E-04 | 2.36E-02 |
| TET1 | -0.47 | 0.72 | 2.75E-04 | 2.42E-02 |
| ADPGK | 0.35 | 1.27 | 2.83E-04 | 2.43E-02 |
| RIOK3 | -0.44 | 0.74 | 2.81E-04 | 2.43E-02 |
| MAN1A1 | -0.99 | 0.50 | 2.94E-04 | 2.48E-02 |
| PEBP1 | -0.62 | 0.65 | 3.04E-04 | 2.54E-02 |
| SLC25A5 | 0.48 | 1.40 | 3.16E-04 | 2.56E-02 |
| TSPYL2 | -0.75 | 0.60 | 3.42E-04 | 2.71E-02 |
| VMP1 | 0.60 | 1.52 | 3.89E-04 | 3.01E-02 |
| GSN | -0.60 | 0.66 | 4.16E-04 | 3.10E-02 |
| DCAF6 | -0.22 | 0.86 | 4.34E-04 | 3.10E-02 |
| MCCC2 | -0.89 | 0.54 | 4.58E-04 | 3.14E-02 |
| CARMIL1 | -0.55 | 0.68 | 4.51E-04 | 3.14E-02 |
| ARHGAP10 | -0.47 | 0.72 | 4.57E-04 | 3.14E-02 |
| ENAH | 0.53 | 1.44 | 4.97E-04 | 3.31E-02 |
| AEBP1 | 0.79 | 1.73 | 4.98E-04 | 3.31E-02 |
| DNHD1 | -0.51 | 0.70 | 5.53E-04 | 3.53E-02 |
| CYP3A5 | -1.40 | 0.38 | 5.74E-04 | 3.62E-02 |
| SCRG1 | -0.89 | 0.54 | 5.87E-04 | 3.64E-02 |
| EIF4EBP1 | 1.06 | 2.09 | 6.04E-04 | 3.71E-02 |
| MBIP | -0.40 | 0.76 | 6.10E-04 | 3.71E-02 |
| OS9 | -0.33 | 0.80 | 6.48E-04 | 3.91E-02 |
| SERINC3 | -0.32 | 0.80 | 6.54E-04 | 3.91E-02 |
| BBS10 | -0.57 | 0.67 | 6.83E-04 | 3.93E-02 |
| CST3 | -0.74 | 0.60 | 6.77E-04 | 3.93E-02 |
| EHD3 | 0.42 | 1.34 | 6.75E-04 | 3.93E-02 |
| SOCS3 | 0.78 | 1.71 | 7.07E-04 | 3.93E-02 |
| ERICH1 | -0.46 | 0.73 | 7.07E-04 | 3.93E-02 |
| TTYH3 | 1.05 | 2.08 | 7.27E-04 | 3.97E-02 |
| HEXB | -0.46 | 0.73 | 7.79E-04 | 4.11E-02 |
| CD9 | -0.80 | 0.57 | 7.82E-04 | 4.11E-02 |
| PTMA | 0.49 | 1.40 | 8.00E-04 | 4.17E-02 |
| SOCS5 | -0.40 | 0.76 | 8.85E-04 | 4.45E-02 |
| GGT7 | -0.54 | 0.69 | 8.90E-04 | 4.45E-02 |
| RPS12 | 0.26 | 1.19 | 9.15E-04 | 4.54E-02 |
| PPP1R3C | -0.71 | 0.61 | 9.54E-04 | 4.68E-02 |
| TPST1 | 0.36 | 1.28 | 1.04E-03 | 4.71E-02 |
| MN1 | -0.80 | 0.58 | 1.04E-03 | 4.71E-02 |
| SNED1 | 0.87 | 1.83 | 1.04E-03 | 4.71E-02 |
| FADS1 | -0.65 | 0.64 | 1.04E-03 | 4.71E-02 |
| ITGA10 | -0.47 | 0.72 | 9.98E-04 | 4.71E-02 |
| SGSM2 | -0.87 | 0.55 | 1.01E-03 | 4.71E-02 |
| ESYT2 | -0.18 | 0.88 | 9.79E-04 | 4.71E-02 |
| MCM3 | 0.39 | 1.31 | 1.01E-03 | 4.71E-02 |
| PRKAG2 | -0.51 | 0.70 | 1.01E-03 | 4.71E-02 |
| ANGPTL5 | -1.18 | 0.44 | 1.11E-03 | 4.84E-02 |
| AFF1 | -0.39 | 0.76 | 1.09E-03 | 4.84E-02 |
| MMP13 | 2.38 | 5.19 | 1.13E-03 | 4.84E-02 |
| PDK4 | -0.88 | 0.54 | 1.13E-03 | 4.84E-02 |
| CCNB1IP1 | -0.33 | 0.80 | 1.14E-03 | 4.84E-02 |
| COL9A3 | -1.62 | 0.32 | 1.16E-03 | 4.89E-02 |

Legend: Log2FC, log2 fold change; FC, fold change; FDR, False discovery rate

**Supplementary Table S7. Previously reported OA risk loci present in our DE gene dataset.**

|  | **Control versus 65%MS cartilage (n=14/group)** | | **Lesioned versus Preserved OA cartilage (N=35/group)** | | **Risk Loci (GWAS)** | | |
| --- | --- | --- | --- | --- | --- | --- | --- |
| **Gene name** | **FC** | **FDR** | **FC** | **FDR** | **Risk SNPs** | **OR** | **Suggested mechanism** |
| *TNC* | 2.80 | 8.51E-03 | 1.41 | 1.09E-02 | rs13321, rs2480930 and rs1330349 | 1.09 | Allelic expression imbalance (AEI) |
| *SCUBE1* | 0.53 | 4.36E-02 | 0.42 | 2.15E-06 | rs528981060 | 1.68 |  |

Legend: Log2FC, log2 fold change; FC, fold change; FDR, False discovery rate

**Supplementary Table S8. All insulin growth factor binding proteins (IGFBPs) and related DE genes identified in our analysis.**

| **Gene name** | **log2FC** | **FC** | **pvalue** | **FDR** |
| --- | --- | --- | --- | --- |
| IGFBP4 | 1.38 | 2.59 | 5.44E-04 | 3.50E-02 |
| IGFBP5 | 2.59 | 6.01 | 3.54E-05 | 7.81E-03 |
| IGFBP6 | -2.37 | 0.19 | 4.72E-08 | 3.07E-04 |
| IGFBP7 | -1.24 | 0.42 | 2.05E-06 | 1.79E-03 |
| HTRA1 | 0.95 | 1.93 | 3.17E-04 | 2.56E-02 |
| STC2 | -1.07 | 0.48 | 1.39E-04 | 1.53E-02 |
| ADAM12 | 0.62 | 1.54 | 6.63E-04 | 3.92E-02 |

Legend: Log2FC, log2 fold change; FC, fold change; FDR, False discovery rate

**Supplementary Figures**

**
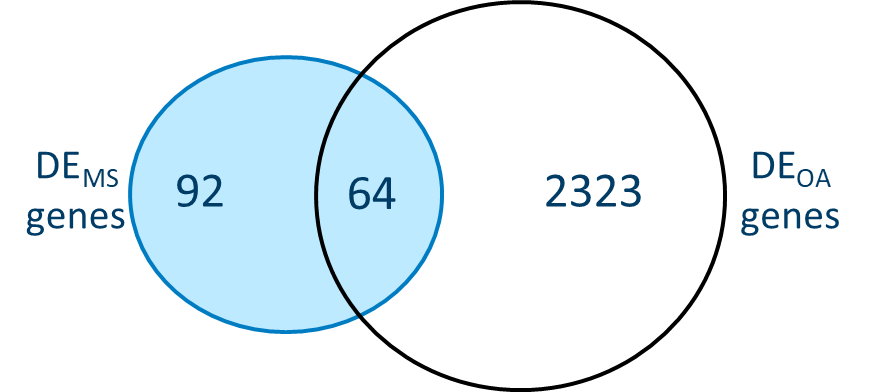
Supplementary Figure S1.** Venn diagram of coinciding genes between differentially expressed genes in mechanically stressed versus control cartilage from osteochondral explants (DE_MS_) and previously identified differentially expressed genes in preserved versus lesioned OA cartilage (DE_OA_) [1].


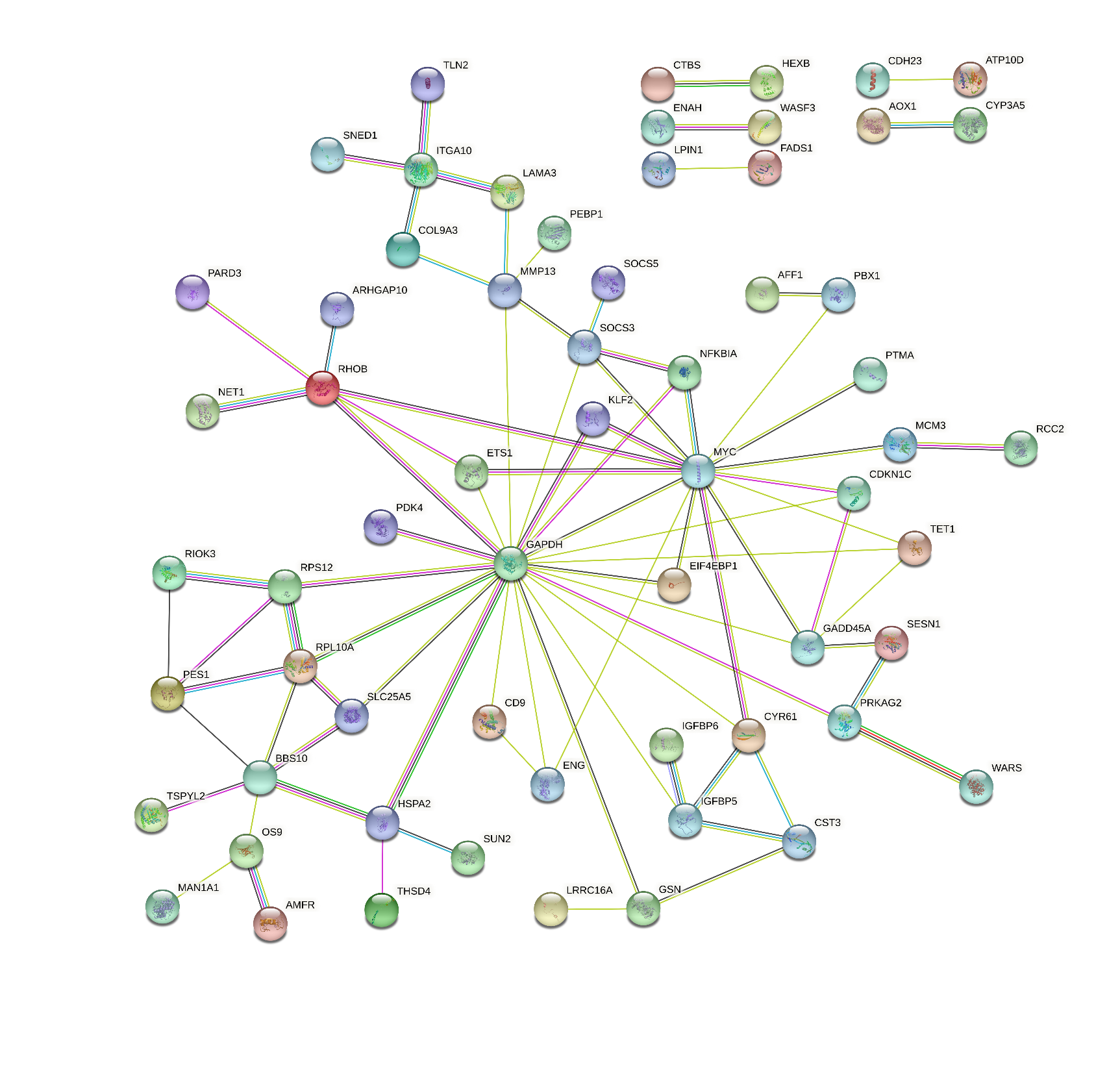


**Supplementary Figure S2.** Protein-protein interaction network in STRING of proteins encoded by differentially expressed genes (N=92 genes) not coinciding with OA pathophysiology (DE_ExclusiveMS_).

**Supplementary Figure S3. Heat-map of proteins present in SASP.** The heat-map depicts the log2 fold change (Log2FC) of gene expression changes in response to mechanical stress (Cart MS; first column) and proteins changes to several senescence inducing treatments found in the SASPatlas [2]. Abbreviations: cart, cartilage; MS, Mechanical stress; Fibro, fibroblasts; IR, X-irradiation; RAS, oncogenic RAS overexpression; ATV, atazanavir treatment; Epi, epithelial.

**References**

1. Coutinho de Almeida R, Ramos YFM, Mahfouz A, et al. RNA sequencing data integration reveals an miRNA interactome of osteoarthritis cartilage. *Annals of the rheumatic diseases*. 2019;78(2):270-7.

2. Basisty N, Kale A, Jeon OH, et al. A proteomic atlas of senescence-associated secretomes for aging biomarker development. *PLoS Biol*. 2020;18(1):e3000599.
